# Development of a Web-Based Acceptance and Commitment Therapy Intervention to Support Lifestyle Behavior Change and Well-Being in Health Care Staff: Participatory Design Study

**DOI:** 10.2196/22507

**Published:** 2020-11-30

**Authors:** Menna Brown, Nic Hooper, Parisa Eslambolchilar, Ann John

**Affiliations:** 1 Swansea University Medical School Swansea University Swansea United Kingdom; 2 Department of Health and Social Sciences University of the West of England Bristol United Kingdom; 3 School of Computer Science and Informatics Cardiff University Cardiff United Kingdom

**Keywords:** participatory design, eMental health, engagement, acceptance and commitment therapy

## Abstract

**Background:**

Positive emotional well-being is associated with healthier lifestyle choices and overall health function, whereas poor mental health is associated with significant economic and psychological costs. Thus, the development of effective interventions that improve emotional well-being is crucial to address the worldwide burden of disease.

**Objective:**

This study aims to develop a web-based emotional well-being intervention for use by health care staff using participatory design to consider adherence and engagement from a user perspective.

**Methods:**

A 3-staged iterative participatory design process was followed, including multiple stakeholders: researchers, computer scientists, mental health experts, and health care staff. Stage 1 used document analyses, direct observation, and welcome interviews; stage 2 used focus group discussions, rapid prototyping, and usability tasks; and stage 3 evaluated a high-fidelity prototype.

**Results:**

Different health care staff (N=38) participated during a sustained period. A structured, sequential, automated, 12-week, web-based emotional well-being intervention based on acceptance and commitment therapy was developed. Freely navigated psychoeducational resources were also included.

**Conclusions:**

The iterative and collaborative participatory design process successfully met its objectives. It generated an in-depth understanding of well-being within the workplace and identified barriers to access. The 3-staged process ensured that participants had the opportunity to explore and articulate criteria relevant to their roles over time and reflect on decisions made at each stage.

## Introduction

### Background

The role and importance of mental health on physical health outcomes, lifestyle behavior, and overall health status has received global recognition [1]. Poor mental health is associated with increased mortality, increased prevalence of physical health conditions, and poorer lifestyle behaviors [2], whereas positive emotional well-being is associated with healthier lifestyle choices and overall better health function [3]. In the United Kingdom, the related economic burden is reported to cost employers between £34.9 (US $45.5) and £45 (US $58.7) billion per year [4,5]. Economic analysis has therefore suggested that investment in staff well-being will lessen this financial impact through reduced absenteeism and staff turnover [5]. Workplaces offer a vital opportunity for health promotion and early intervention to improve poor health-related lifestyle behaviors and are appropriate places to provide well-being resources and support. Equally, public sector staff has higher sickness and absenteeism rates than private sector staff [6]. Thus, the development of effective interventions that improve mental health and emotional well-being is crucial in addressing the disease burden in this population.

### Web-Based Approach

Web-delivered interventions, which are understood to be effective and cost-effective, may be helpful in this regard [7-12]. However, poor adherence and engagement remain a critical concern that limits treatment outcomes [13]. This is particularly the case for open access programs in which adherence has been as low as 3% [14]. Many avenues have been explored to address this issue, including interactive design features [15,16], persuasive technology [17], social interaction [18], rideshare services [19], and gamification [14].

### Participatory Design

This study seeks to describe the development of a web-based emotional well-being intervention using participatory design (PD). We adopted this approach to improve adherence and engagement. PD is a collaborative process that includes anticipated end users in the development of new products, uses diverse research methods such as qualitative inquiry, and has the potential to offer critical insight and understanding of users’ motivation and engagement in such interventions [20]. Informed by action research, PD has seen rapid growth in popularity and application across diverse fields, including commercial product design, industrial design, architectural design, and government space programs [21]. PD has also been incorporated in several health care contexts [22-25], including youth mental health [26,27] and dementia care, or mental health care for older adults in general [28,29].

Findings from this diverse literature have highlighted that end-user input can be incredibly useful. Specifically, the involvement of anticipated end users, not unlike the *expert patient* role in the psychological literature, can highlight, early on, key information regarding user needs, understanding, knowledge, and values that can support the development of effective resources [23]. In addition, active user involvement across health care research has been widely promoted in patient settings and continues to be of critical importance. For example, the Patient and Public Participation policy 2017 [30] set out the National Health Service (NHS) England’s commitment to strengthening user involvement in service design and delivery, and the National Institute for Health and Care Excellence [31] has published similar sentiments.

Although PD has been used to investigate well-being in other contexts and has reported positive findings, [32,33], we are unaware of any study that has used PD in the development of a web-based emotional well-being intervention for staff use within a health care setting, with the underlying purpose of addressing adherence and engagement.

### Objectives

We aim to develop a web-based emotional well-being intervention for use by health care staff using PD, with specific objectives of exploring the following:

1. The workplace context, access and availability of existing resources, and workflows;
2. Understanding of well-being in a workplace context;
3. Therapeutic approach;
4. Website design (style, logo, and layout);
5. Interactive and access features (structure, gamification, and audio or visual components);
6. The aforementioned concerning adherence and engagement;
7. Identification of criteria relevant to participants.

## Methods

### Ethics

Ethical approval was provided by the Swansea University Human and Health Research ethics committee (July 2015). Abertawe Bro Morgannwg University Health Board (ABMU HB) granted approval for service development.

### Participants

Participants were staff from a Welsh health board (HB) in the United Kingdom. There are 7 HBs in Wales that serve a total population of 3.2 million people; each HB includes hospitals, outpatient clinics, and general practices. Staff was invited via intranet, email, and presentation. Digital and physical notice boards displayed the study flyer.

### Inclusion Criteria

The inclusion criteria were being a member of staff at the selected HB and age ≥18 years.

### Procedure

**Figure 1:**
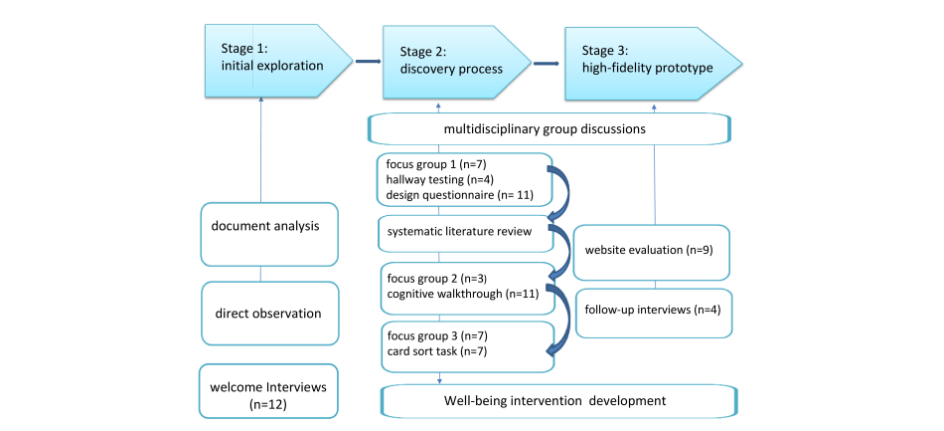
Study diagram.

The PD process followed 3 distinct stages (Figure 1) specifically combined to elicit a progressive design process [34] in line with the ISO (International Organization for Standardization) 134407 (1999) [35] standards of human-computer design.

#### Stage 1–Initial Exploration

We conducted the identification and exploration of organizational work environments (Table 1). Workers’ routines, day-to-day functions, traditions, and practices alongside the availability of existing (workplace) health and well-being resources informed the understanding of how and when staff might access the intervention [36].

**Table 1:** Data sources.

Sources of evidence	Description	Data collected
Document analysis	ABMU HB^a^ public website Published publicly available reports from ABMU HB Publications from phases I and II of Champions for Health^b^ Public Health Wales evaluation documents of Champions for Health Well-being through work service	Major and local hospital sites in ABMU HB Staff roles within the health board Employment statistics Champions for Health development Evaluation of past campaign results (phase I): engagement, retention, and adherence Profile of typical staff member who took part Health improvement rate Identification of current well-being resources available via the service
Direct observation	Visit to main hospital sites	Observations of physical space, organization, and use
12 open-ended welcome interviews	Staff from a variety of roles within ABMU HB	Description of the design process Discussion of the role in the design process Clarify understanding and requirements of participation Explore motivation for participation Explore previous experiences of PD^c^ Explore initial thoughts on well-being Administer a questionnaire to explore access to internet-capable devices and the current work environment Description of benefits and difficulties of participation Clarify the ability to attend focus groups

^a^ABMU HB: Abertawe Bro Morgannwg University Health Board.

^b^Champions for Health was a health promotion platform developed by Public Health Wales, which consisted of 5 lifestyle behavior change modules (quit smoking, alcohol reduction, weight optimization, regular exercise, and eat healthily) for use by health care staff in Wales, United Kingdom.

^c^PD: participatory design.

#### Stage 2–Discovery Process

In line with accepted traditions [37], this stage focused on study objectives 2 to 7 via clarification of participants’ values and tacit knowledge through continuous and cooperative interaction with multiple stakeholders [37], focus group discussion, and rapid prototyping [38,39].

##### Focus Groups

The initial focus group (FG1) included the principal researcher (MB) and anticipated end users. The group explored participants’ goals and values, with the purpose of generating a shared project plan, which specified well-being needs that could or should be met by the intended resource. Participants’ understanding of well-being in the context of their workplace was explored in-depth. Thereafter, a discussion was held, which focused on evaluating existing websites (eg, *MOODGYM* and *Color Your Life*). This generated initial design ideas and identified likes, dislikes, key website features, intervention content [36], and therapeutic approach. Ideas from the welcome interviews were presented in the form of a word cloud to generate further discussion.

New members (anticipated end users) were included in subsequent focus groups, in line with accepted recommendations [34], as were 2 computer scientists. Their insight supported discussion on interactive features and design guidelines and ensured that a variety of perspectives were incorporated [36]. To promote and strengthen the relationship between researchers and participants, focus groups were held at a variety of hospital locations [37]. Focus group 2 (FG2) recapped the project plan, undertook a data validation exercise based on FG1 outcomes, and explored options for the therapeutic approach. Focus group three (FG3) explored content requirements, including structure, gamification elements, and audio or visual features. Gamification elements included health points and trophies as a reward for website engagement and feedback graphs to show progress. This stage was also informed by 2 systematic literature reviews [14,40].

##### Rapid Prototyping

Regular multidisciplinary group discussions were held to discuss and interpret the data. Low-fidelity prototypes (Multimedia Appendix 1) were produced to attend to cost and time considerations, stimulate early design discussions [37], and identify design errors early, in line with the 5 essential processes of human-centered design principles [35]. Rapid prototyping was undertaken after each focus group in an iterative cycle, and feedback informed subsequent designs.

##### Hallway Testing

Individuals in an office setting were randomly approached and asked to participate. The office setting was selected because of its environmental similarity to the anticipated end-user context. Context is considered a key factor in the development of web and mobile apps [41,42]. Participants were briefed and debriefed. Layout ideas were explored using paper prototypes.

##### Design Task

**Figure 2:**
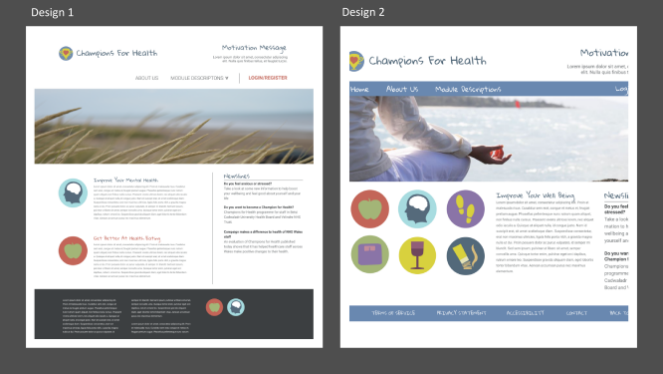
Home page designs version 1 and 2 used in stage two.

Visual aesthetic appeal is of critical importance in web design [43-46]. For example, empirical findings suggest that individuals reach a decision regarding the visual appeal within 50 milliseconds [46]. The design task was conducted in a group setting, in a large room with a large whiteboard at the front, with a clear view. Participants were briefed and administered a printed questionnaire. A researcher displayed designs on the whiteboard for 7 seconds. Participants rated each design using a 5-point Likert scale ranging from 1=very unappealing to 5=very appealing; 2 sets of logo designs were also shown, and preference was indicated (Figure 2). Once completed, the designs were discussed, and written feedback was provided.

##### Cognitive Walkthrough

The cognitive walkthrough was selected for its ability to generate data regarding typical user responses to designs and navigation. Testing the initial designs in this way ensured that development remained focused on user experience, interaction, and responses [44]. However, exploration and evaluation were limited to the use of simple designs and were not assumed to be indicative of response times.

Participants’ verbal responses and interactions with the *system* were video recorded. A video camera was set up on a tripod behind the participant to capture hand movements while navigating the prototype. Initial website designs were prepared beforehand for the task, and designs were printed out in full color. Participants were asked to “imagine themselves in their usual work environment, with some time available to explore on-line, their interest in their own health and well-being.” One researcher asked a series of realistic questions, and another acted as the *system*, responding according to user behavior. For example, the home page was presented, and participants were asked to select a well-being resource they would like to explore. They showed their selection by *pretending* to click on the website buttons available. Depending on their selection, the *system* then presented the next screen (ie, if they selected *improve your well-being,* this led to the *yoga girl* page. Alternatively, if they selected *module description*, a drop-down menu appeared). A third researcher took field notes.

##### Card Sort Task

An audio-recorded, open card-sorting task informed the organization of content and created category labels. Participants worked collaboratively. The group approach was selected because of staff time constraints. Participants were briefed and debriefed and provided with a set of 25 cards with the following categories: acceptance and commitment therapy (ACT), mindfulness, acceptance, cognitive fusion, being present, self as context, committed action, values, relaxation exercises, benefits of relaxation, sleep hygiene, sleep and well-being, sleep diary, photo gallery, map, restorative effects of nature, symptoms of stress, symptoms of anxiety, symptoms of depression, what is stress, what is anxiety, what is depression, ACT exercises, whom to contact, and homework. Participants were asked to categorize the cards as they saw fit. No limit was placed on the number of categories available. Once all the cards were sorted into categories, participants were asked to discuss among themselves the possible labels for each of the categories identified. Options were written down on blank cards and discussed to reach consensus.

##### Intervention Development

The iterative development process among researchers, computer scientists, and participants was mirrored with an iterative intervention development process. The primary researcher compiled a draft informed by data from stages 1 and 2. This was discussed and reviewed by a mental health expert and an expert ACT practitioner who provided detailed feedback. This ensured that the content was developed in line with therapeutic principles, incorporated appropriate scenarios and examples, outlined key concepts clearly, and supported positive well-being.

#### Stage 3–High-Fidelity Prototype

A high-fidelity prototype website, informed by data from stage 2, was developed (Multimedia Appendix 2), and therapeutic content was added. Participants accessed a private WordPress website for 6 weeks. Participants provided consent by actively visiting the website and requesting access. A blog update was posted to the website once a week, which served as a reminder to access the website. Structured feedback was requested on completion of each week via anonymous embedded surveys. Alternative feedback routes were available: website blog and direct email. At the end of week 6, a debrief message was posted, and the website was closed. All users were invited to participate in an interview to discuss their experiences. Users completed 2 validated self-report measures before accessing the website, the World Health Organization Well-Being Index (WHO-5) [47] and the Acceptance and Action Questionnaire version II (AAQ-II) [48], an assessment of psychological flexibility.

### Data Analysis

#### Interview and Focus Group Data

Interviews and focus groups were audio-recorded and transcribed verbatim. Inductive thematic analyses were performed, informed by the work of Braun and Clark [49], where a staged process of data analysis was followed: familiarization with the data, reading and rereading of transcripts, and immersion in the data set, which was achieved through an active process of memoing keywords, trends, and recurring patterns observed in the data. Initial codes were then generated using a line-by-line approach, summarizing the data to capture the essence of participants’ thoughts and views. This was followed by reporting the codes in a formal coding structure document. Stage 3 involved searching for themes across the data set using the coding structure and thematic mapping of codes and emergent concepts. A full review of themes and codes was undertaken in stage 4, followed by refinement and development of theme names. Throughout the process, emergent themes were discussed with a second researcher. Participant quotes and extracts were highlighted, and theme development memoing was undertaken using a constant comparative method to ensure all data were included for analysis and interpretation.

#### Prototype and Usability Data

##### Hallway Task

A multidisciplinary group discussed feedback. Design questionnaire data frequencies were reported, and free-text comments were qualitatively analyzed.

##### Cognitive Walkthrough

Data were analyzed in a structured manner [44]. First, the video-recorded data were watched, and question responses transcribed verbatim to capture verbal and physical responses. Second, the data were scrutinized for error frequency and type [44]. A correct response was indicated by a score of 0 and an incorrect response by a score of 1 and a full description. This was undertaken for each task question. A response was considered correct if it met the expected user action for that question. For example, when asked, “What would you do if you were interested in finding out about emotional well-being?” the expected responses would include “click on the well-being icon” or “navigate to the drop-down menu, explore modules and click on emotional well-being option.” All potential navigation routes were identified. An incorrect response would be any other answer, for example, “click on the five a day icon or option in the drop-down menu.” Finally, incorrect responses were assessed and assigned a risk score. A summary document was produced, which categorized all incorrect responses and risk assignment and highlighted critical incidents.

##### Card-Sorting Task

Categories and subcategories labeled by the group were reported.

##### High-Fidelity Prototype

Engagement was measured by weekly survey completion.

## Results

Results are presented per PD stage; in stage 1, document review data are presented only on HB.

### Stage 1

#### Document Review

At the time of the study, ABMU HB employed 16,000 staff and served a population of approximately 6000,000 with an annual budget of £1.3 billion (US $ 1.70 billion; ABMU annual report 2010-2011). The HB consisted of 4 acute hospitals, 10 community hospitals, and 77 general practices. Within the NHS Wales and ABMU HB, anxiety, stress, depression, and other unspecified psychiatric illnesses affected 7945 staff members (ABMU HB report) in 2015 and 2016 and accounted for 23% of sickness absences in 2015, second only to musculoskeletal conditions (25%). Employee well-being (occupational health) consisted of clinically led well-being through work service. Later (2016), a voluntary staff *Well-being Champions* scheme and an annual staff well-being week (2017) were introduced alongside a range of informal local initiatives (eg, running club and book club).

#### Welcome Interviews

A total of 12 welcome interviews were conducted between August 8, 2015, and September 23, 2015, across a variety of locations. Interview duration ranged between 30 and 60 min, and 83% (10/12) of the participants were women aged 31 to 60+ years (Table 2). Staff was from a range of occupations: consultant, physiotherapists, occupational therapists, speech therapists, administration, education, and managerial staff.

Interview data identified that all participants had access to an internet-enabled computer device during their working day, and 10 also had Wi-Fi access. However, access restrictions varied, and some were limited to 30 to 45 min of personal use during break time. Initial resource ideas were identified and presented as a word cloud to generate discussion in FG1 (Multimedia Appendix 3). A key issue that emerged was related to how individuals managed the constant changes at the organizational level, which affected health and well-being. This theme was then explored further in FG1.

**Table 2:** Participant data.

Stage and task		Date	Location	Duration (min)	Number of participants	Number of women, n (%)	Age range (number)
**Stage 1**							
	Welcome interviews	August 11 to September 23, 2015	Multiple locations^a^	30-60	12	10 (83)	31-40 (4), 41-50 (4), 51-60 (3), and 60+ (1)
**Stage 2**							
	Focus group 1	September 28, 2015	Princess of Wales Hospital	120	7	5 (71)	31-40 (1), 41-50 (3), 51-60 (2), and 60+ (1)
	Focus group 2	December 8, 2015	Princess of Wales Hospital	60	4	1 (25)	21-30 (1), 31-40 (2), and 60+ (1)
	Focus group 3	March 21, 2016	Neath Port Talbot Hospital	60	7	4 (57)	31-40(1), 41-50 (3), 51-60 (2), and 60+ (1)
	Hallway task	October 5, 2015	Swansea University singleton park campus	—^b^	4	—	—
	Design task	October 12, 2015	Singleton Hospital	—	11	—	—
	Cognitive walkthrough	February 26, 2016	Singleton Hospital	—	7	—	—
		December 8, 2015	Princess of Wales Hospital	—	—	—	—
	Card sort	March 21, 2016	Neath Port Talbot Hospital	—	7	—	—
**Stage 3**							
	High-fidelity website	October 1, 2018	N/A^c^	6 weeks	9	—	—
	Follow-up interview	December 3, 2018- January 9, 2019	Neath Port Talbot Hospital, Princess of Wales Hospital, Morriston Hospital, and Singleton hospital	12-30	4	3 (75)	31-40 (4)

^a^A variety of locations across ABMU HB and Swansea University singleton campus.

^b^Not collected.

^c^N/A: not applicable.

### Stage 2

A total of 38 different staff members participated; some took part in multiple tasks.

#### Focus Groups

Following the welcome interviews, 5 female participants decided not to take part in the subsequent stages of the project. One was unable to travel to attend a focus group, another changed roles, and 3 others had limited availability to participate; 9 additional participants were recruited (2 participants from FG1 also attended FG2).

A total of 3 focus groups were conducted (n=18) at 2 hospital locations between September 28, 2015, and March 21, 2016; 56% (10/18) were women, and the duration of discussion ranged from 60 to 120 mins. Participants were aged between 21 and 60+ years (Table 2) and from a variety of professions: education, speech therapy, physiotherapy, nursing, occupational therapy, and management.

A shared project plan was created based on discussions of participants’ goals and values (Multimedia Appendix 4). Discussion of participants’ views and understanding of well-being led to the emergence of 5 themes (FG2 and FG3 data validation process did not identify any new themes) that directly shaped the emergent intervention: meaning, causes of poor well-being, well-being as taboo, well-being needs and barriers, and resource suggestions (Multimedia Appendix 5: Themes). For example, well-being was broadly described as a sense of life balance and positive living across different life domains: work or career, family, personal and social life, and took into account aspects of enjoyment, responsibility, and choice and ability to pursue activities in a domain without feeling restricted by responsibilities in other domains. As such, the intervention resources needed to reflect this perspective. Equally, attention was paid to causes of poor well-being at the individual level, and as such, the intervention needed to focus on encouraging a growth mindset to address perceived poor self-awareness and enable users to self-manage their own well-being through the provision of interactive ideas and information. Well-being was considered a taboo workplace topic, and it was felt that the resources needed to reach across the organization to ensure all staff was included. As a result, individual profiles (for different staff groups) were not included to give a sense of cohesion or commonality.

Participants openly discussed their experiences with different therapeutic approaches, some from the educator’s role, others from a more personal perspective. There was a collective sense that something new was needed, a divergence from existing knowledge, and the need to not duplicate existing resources available within the HB. During discussions, a range of approaches was presented; participants identified that cognitive-behavioral therapy was already available and widely used and that an alternative approach would be beneficial. ACT emerged as an appropriate choice; several participants had experience of ACT and believed it was not too out of step with staff experiences despite being less well known. The key focus was on creating a resource that would promote positive well-being on a day-to-day basis and could be relied upon in times of emotional difficulty.

Stress management was a central focus and a range of resources and interactive content were identified for inclusion, for example guided mindful meditations, Tai Chi and breathing exercises, and experiential exercises (a key feature of the ACT model) supplemented with tailored scenarios to (nonspecific) health care staff needs and downloadable activities. Likewise, personal shared stories and the need for clear signposting were identified as important to help staff understand their personal experiences and identify onward sources of support. This resulted in the inclusion of 5 well-being films (Multimedia Appendix 6) and a list of outward organizations.

Equally, alongside content options, intervention features and organization were discussed. Participants were happy with a sequential format because of its ease of use and familiarity, but they wanted different progress routes to recognize the different professional groups working across the HB, for example, the stress and strains experienced by professions and the different access and time options available. Therefore, additional psychoeducational materials were included. This would also attend to the needs of those looking for a *quick fix* on the understanding that not all users would need or want a long-term approach. Audio and visual communication was preferred over long text sections. A mobile responsive website was also a key requirement. Blogs and chat rooms were discussed, and their relative merits were considered with regard to the feasibility of programming and privacy issues informed by computer scientists.

#### Rapid Prototyping

We undertook 4 prototyping and usability tasks (n=33) at 4 locations between October 5, 2015, and March 21, 2016. Demographic data were not collected.

#### Hallway Testing and Cognitive Walkthrough

The hallway testing data were combined with low- (paper designs used) and high-fidelity cognitive walkthrough data. We identified 3 key issues: first, user difficulty navigating away from the *pop-up* pages (nature, sleep, and relaxation pages); second, user uncertainty regarding the use of the resource; and third, confusion over expectations of the *nature page*. As such, an additional close button was added to each pop-up, instructions for use were incorporated into the registration page (to guide users), and the pop-up was renamed *Green space*.

#### Design Questionnaire

**Figure 3:**
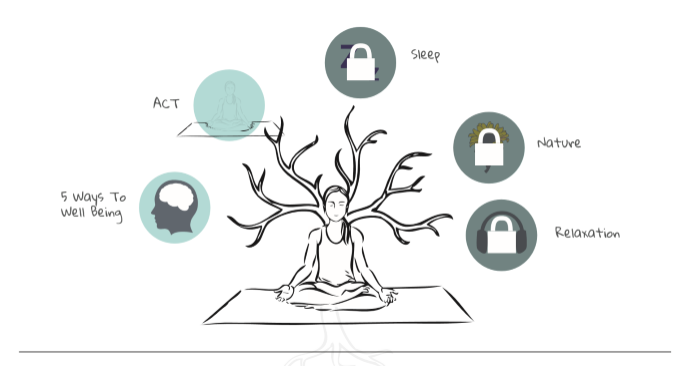
Initial well-being design.

Design data informed the color scheme, module logos, well-being design (Figure 3), and home page layout (design 2 was selected).

#### The Card Sort

We identified 8 categories, labels or headings, and subcategories. Key outcomes were category labels, the requirement for additional information on stress, depression, anxiety, and integration of resources throughout the module.

### Stage 3–High-Fidelity Prototype

In this stage, 2 new participants were included. A total of 9 participants piloted the high-fidelity intervention for a period of 6 weeks (October 2018). No demographic data were collected. Four participants completed a follow-up interview, conducted between December 3, 2018, and January 9, 2019, at 4 hospital sites. Most of them were women (3/4, 75%), aged 31 to 40 years, and from the following professions: therapy, pharmacy, physiotherapy, and occupational therapy. The interview duration was between 12 and 30 mins.

A total of 7 participants completed the WHO-5 questionnaire, and 8 completed the AAQ-II before accessing the website. Engagement with the intervention content varied; 1 participant remained engaged until week 5, although the interview data indicated that the content across all 6 weeks was viewed. The survey responses indicated that overall, the content was considered useful and contained adequate information. Moreover, the responses indicated that most interactive content (ie, experiential exercises, YouTube clips, and *try now* activities) was explored and that the *lesson summary* was helpful.

3 feedback routes were utilized: blog post (n=2), email (n=2), and handwritten feedback (n=1; Table 3). Written feedback and interview data highlighted additional suggestions, including alternative ways to display content, the need to expand descriptions and embed YouTube clips, the use of audio files as an alternative to text, and inclusion of *time to complete* estimates for each experimental exercise and interactive element. Barriers to use were a lack of time (during the working day) to access the website, lack of internet access via workplace computers, no headphones for audio components, perceived lack of managerial support, and volume of text content. Interview participants discussed the length of time they felt they had spent each week and how this might be reduced or broken down into shorter, more manageable segments to encourage engagement.

**Table 3:** Summary of key survey results and free-text feedback.

Week	Number of respondents, n (%)	Overall usefulness of week, 5-point Likert scale (number of responses)	Participants who completed interactive content, n (%)	Free-text comments (feedback route)
1	6 (67)	Useful (3) Somewhat useful (1) No response (2)	4 (44)	“ACT model may need more of an intro/explanation or something like ‘the model will be revisited throughout the programme’ or ‘the 6 techniques will be expanded upon throughout...’” [Blog] “The watch links need some text to contextualize why we are now moving to deep breathing etc as they feel a bit random at the mo.” [Blog] “An explanation at this point [Psychological flexibility questionnaire] on what the scores mean would also be helpful.” [Email]
2	3 (33)	Useful (3) Somewhat useful (1)	3 (33)	“good - week two seems a lot easier to read through and doesn't feel as intense” [Survey] “I liked the really clear language and the great use of examples. The metaphors were well chosen. I wonder about the tiger metaphor though... I did not download PDFs but I used them at the time” [Survey]
3	3 (33)	Very useful (1) Useful (1) Somewhat useful (1)	3 (33)	“I think there were too many exercises this week” [Survey] “In the ending you mention thoughts and feelings as barriers, but not necessarily behaviors. Wondering if it was worth putting that in as all three impact each other.” [Survey]
4	3 (33)	Useful (3)	3 (33)	“Maybe somewhere it could advise on the amount of time needed” [Survey] “The unwanted party guest YouTube example was excellent and in my opinion the most engaging.” [Survey]
5	1 (11)	Useful (1)	1 (11)	“It was quite a short week compared to the others, but I would not have wanted anymore on the particular topic, there was more than enough detail on it.” [Survey]
6	0	No responses	0	“I like the way it’s got the same layout every week it makes it easy to follow” [Interview] “I think people could use it for their CPD time.” [Interview] “I’ve seen things like they tell you how many mins it might take, so this is going to be a 5 mins exercise.” [Interview] “I haven’t been able to access them [YouTube clips] not because of your website but because of our, we are only allowed on certain websites.” [Interview]

### The Intervention

A 12-week, emotional well-being intervention based on ACT was developed in line with participant discussions and systematic review [14,40] outcomes. This was added to the new study website alongside the existing Champions for Health 5 lifestyle modules. ACT [50] is a third-wave therapy that encourages the development of psychological flexibility. Specifically, it aims to equip people with the ability to more skillfully relate to their unwanted thoughts and feelings such that they are still able to move toward personally chosen values. Contrary to other approaches, ACT asks people to be willing to experience negative private events, rather than seeking to change them, and a central theme of the approach is to support people in returning their attention to the present moment through mindful practice.

**Figure 4:**
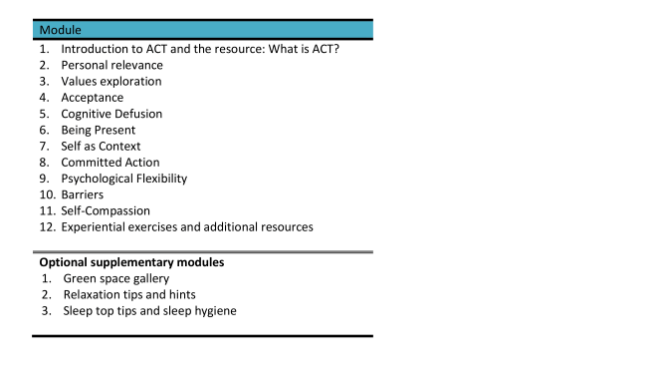
Intervention sequence.

The intervention incorporated 6 core processes of ACT (Figure 4). Each resource was compiled as a *week* and designed to *stand-alone* to encourage repeated use and skill consolidation and avoid overwhelming those new to ACT. Users-identified features that reduced or encouraged engagement were added (Textbox 1).

**Engagement.**

Participant-led suggestions to enhance engagement:

- Short interactive segments
- Time to complete information (ie, estimated amount of time the exercise will need)
- Lesson summary or take-home message
- Management and organizational-level support for workplace use of resources and specified well-being time
- Completion certificate to integrate with organizational-level professional development reviews in recognition of time spent and skills acquired

## Discussion

### Principal Findings

This study aimed to develop a web-based emotional well-being intervention for use by health care staff in the workplace, following a staged PD process. Specific objectives were to explore workplace context, access and availability of existing workplace well-being resources, and anticipate end-user opinion on well-being workplace needs, therapeutic approach, website design, and interactive features while exploring these in reference to promoting user engagement. The process also aimed to identify and explore additional criteria relevant to participants.

A structured, sequential, automated, 12-week, web-based emotional well-being intervention based on ACT was developed as a result of an iterative, 3-staged PD process. A wide range of staff engaged in many activities at different time points during a sustained period. Workforce groups with high- and low-rate absenteeism were represented. Absence due to sickness among ambulance staff, health care assistants, and support staff was 6.3% and 6.2%, respectively, whereas absence among nurses, midwives, health visitors was lower (1.05%), as well as medical and dental staff (1.21%) [51].

The collaborative approach supplemented by recommended mental health design guidelines [41] incorporated qualitative methods of inquiry, facilitating open discussion, and generating a collective codeveloped understanding of well-being in the workplace. Open discussions between researchers and participants created a supportive group environment, evidenced by staff discussions of personal mental health experiences and well-being needs. This enabled topics to be revisited over time, and as new members joined. *Design for outcomes* [41] advocated concise goals and focus on intended outcomes, adhering to this enabled effective management of expectations at each stage and resulted in an immediate array of design suggestions, resources, and the participant-led selection of ACT.

The inclusion of multidisciplinary experts and participants from different workplace environments outlined in “design in collaboration with mental health professionals” [41] provided critical insights from a range of perspectives and allowed issues relating to access, organizational support, and stigma to be considered.

Frequent meetings with computer scientists and rapid prototyping not only stimulated discussions on website style, look, and color scheme but also generated specific design requirements. The design questionnaire, cognitive walkthrough, and card sort enabled quick and accurate identification of frequent user error, unsuitable features, and areas that required further clarification. Participants engaged easily with these tasks, which were selected for ease of use. Similarly, the importance of identifying user needs and being sensitive to mental health state when considering delivery and reminder options to participants [22] was central to the intervention created. Equally, the iterative intervention content development process was informed directly by participants at all stages, while remaining under careful inspection of clinical experts.

Adherence and engagement remain a critical concern in web-delivered mental health interventions. PD was selected specifically to address this issue. Participation in the design and development process is thought to increase the likelihood of user ownership and alignment with the end product [52] while simultaneously affording the opportunity to explore anticipated end-user views on methods to promote sustained engagement and adherence. The continued interest of different participants over a sustained period indicated that this was successfully achieved, despite lower engagement in stage 3. Participants were also asked to suggest ways in which to address poor use in relation to their workplace. Barriers to access (to well-being resources) identified in the qualitative discussions led to a series of communications and a meeting with the executive director of public health at the HB and the establishment of a sustained working relationship with the *employee's well-being* team at the HB. Support at the organizational level, including managerial support, remains an integral component of the project.

### Comparison With Previous Work

Our findings are in line with those of Wadley et al [26], who reported successful use of PD processes to develop a web-based social therapy intervention for adolescents with psychosis. Similar to our approach, they followed a PD process where initial design ideas were presented to patients to stimulate discussions surrounding web-based delivery of therapy and personalized preferences, followed by separate discussions with clinicians.

Kelders et al [16] developed an intervention to prevent depression. They used participant interviews, rapid prototyping, and requirement sessions and concluded that their methods provided valuable insight beyond comments on color and layout and extended to include contextual considerations. Similar to this study, staff considered their workplace context and well-being needs throughout the development process, and this added valuable insight into how and when the program might eventually be accessed and included in daily work schedules. Barriers to use were identified, which facilitated the development of quickfire experiential exercises that specified the expected completion time (ie, 5-min duration). This ensured that the exercise was suitable for workplace use, that is, could be used on a short lunch break or during well-being time, which was supported by management. Management support and *permission* to use the resource were highlighted as critical elements to enable engagement with workplace interventions.

### Limitations

Previous work has noted that PD approaches have been difficult to sustain within complex health care contexts where anticipated end users and stakeholders are busy and cannot commit adequate time to the iterative process of designing [53,54]. Although a range of participants from diverse professional streams worked well together and participated with active and ongoing interest, some were unable to attend successive stages because of busy work schedules, organizational commitments, and varying geographical locations. Therefore, some tasks had small numbers, which limited data analysis. Focus groups were also limited to a maximum of 2 hours. Although this was adequate, longer sessions might have facilitated further insight and design developments, which should be considered thoroughly in future projects of this kind. Future work may consider web-based focus groups and workshops to mitigate this [55].

Stage 3 was limited by a small sample and poor survey response rate; however, interactive elements were used. The final focus group was altered to one-on-one interviews because of conflicting work schedules. This limited exploration of the user experience. Further incorporation and discussion of how participants might use and practice experiential exercises and interactive intervention features could have been introduced into the design process, for example, through *future workshops*, which asks participants to envisage and discuss the future use of the technology [56]. However, mental health experts, who are arguably better placed to review content, conducted a full review of the resources, and contributed to the iterative design process.

Due to time constraints, an assessment of cognitive workload was not undertaken. This is desirable because web-based delivery methods often rely on text to convey complex messages. However, the readability of the main website was assessed and considered to be a grade level 9, meaning it should be easily understood by those aged from 13 to 15 years [57].

### Conclusions

This study brings together strands of public health, psychology, and medicine with computer science to develop an emotional well-being intervention via PD methods.

This study makes two key contributions. First, it offers insights for future practice by presenting empirical data reported from a range of stakeholders. The focus on features to enhance and promote engagement in a workplace well-being resource is of particular interest. Second, it contributes to the developing body of knowledge regarding the utility of the PD approach within the context of health and well-being.

We conclude that the study objectives were met. The PD process successfully facilitated exploration of the anticipated end user’s workplace context, access and availability of existing resources, and existing workflows, and it generated an in-depth understanding of workplace well-being, specifically, barriers to access. Participants selected the therapeutic approach through collaborative discussion and consideration of shared knowledge, understanding, and need. Rapid prototyping led to an iterative participant-led design cycle that identified style, logo and layout requirements, interactive intervention features, and structure. The 3-staged process also ensured that participants had the opportunity to explore and articulate criteria relevant to their roles over time and reflect on decisions made at each stage.

## Appendix

Multimedia Appendix 1 Low-fidelity prototype designs.

Multimedia Appendix 2 High-fidelity prototype designs.

Multimedia Appendix 3 User generated word cloud used in stage two.

Multimedia Appendix 4 Project plan.

Multimedia Appendix 5 Qualitative themes.

Multimedia Appendix 6 PocketMedic well-being films.

## Abbreviations

AAQ-II: Acceptance and Action Questionnaire version II
ABMU HB: Abertawe Bro Morgannwg University Health Board
ACT: acceptance and commitment therapy
FG1: initial focus group
FG2: focus group 2
FG3: focus group 3
HB: health board
NHS: National Health Service
PD: participatory design
WHO-5: World Health Organization Well-Being Index
