# Supplementary figures and images for: Development of a Web-Based Acceptance and Commitment Therapy Intervention to Support Lifestyle Behavior Change and Well-Being in Health Care Staff: Participatory Design Study
Source: JMIR Form Res. 2020 Nov 30;4(11):e22507. doi: 10.2196/22507 (PMC7735901; doi:10.2196/22507)

## Slide 1
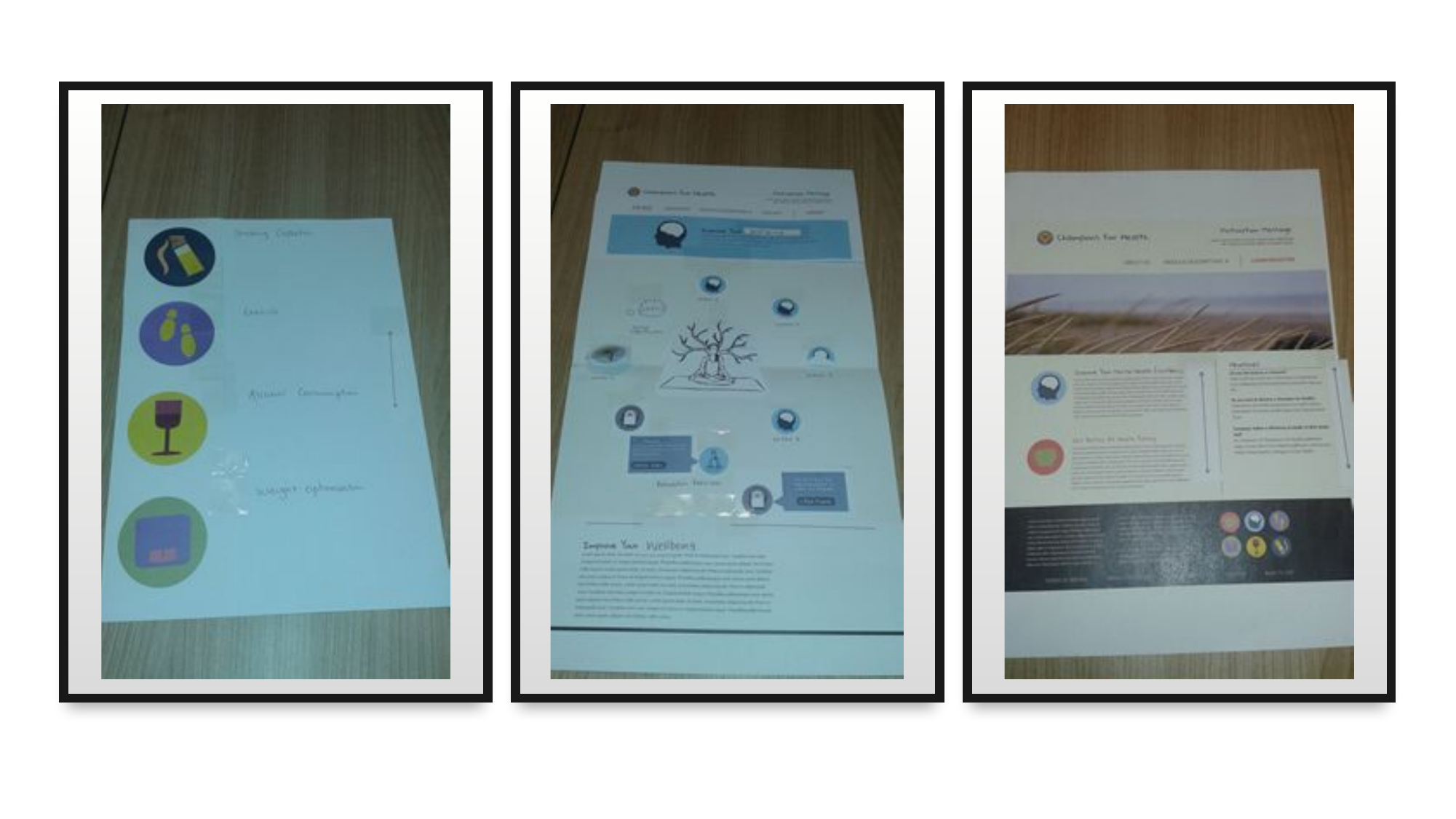

## Slide 2
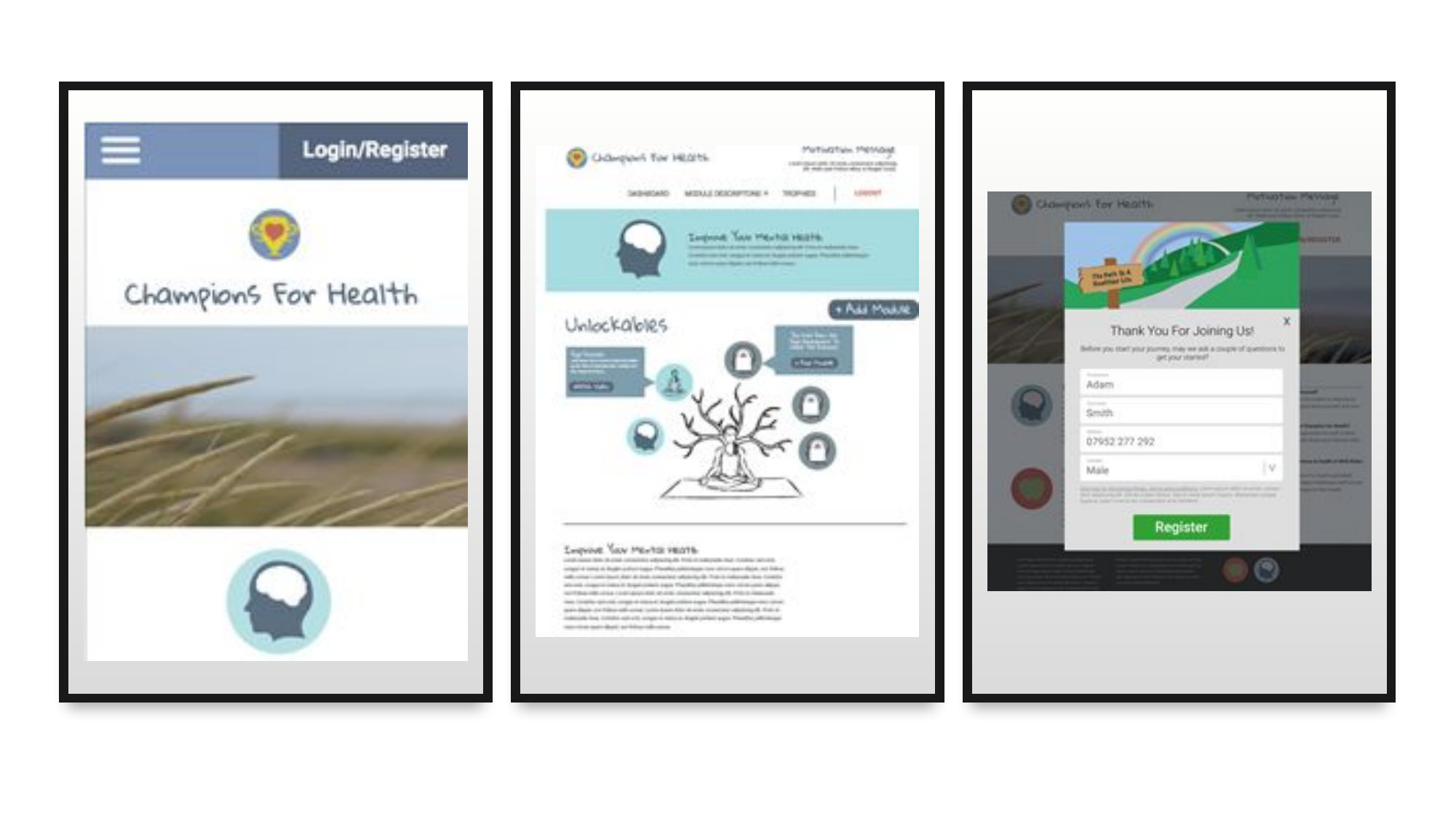

Supplement: Multimedia Appendix 1 [file formative_v4i11e22507_app1.pptx]

## Slide 1
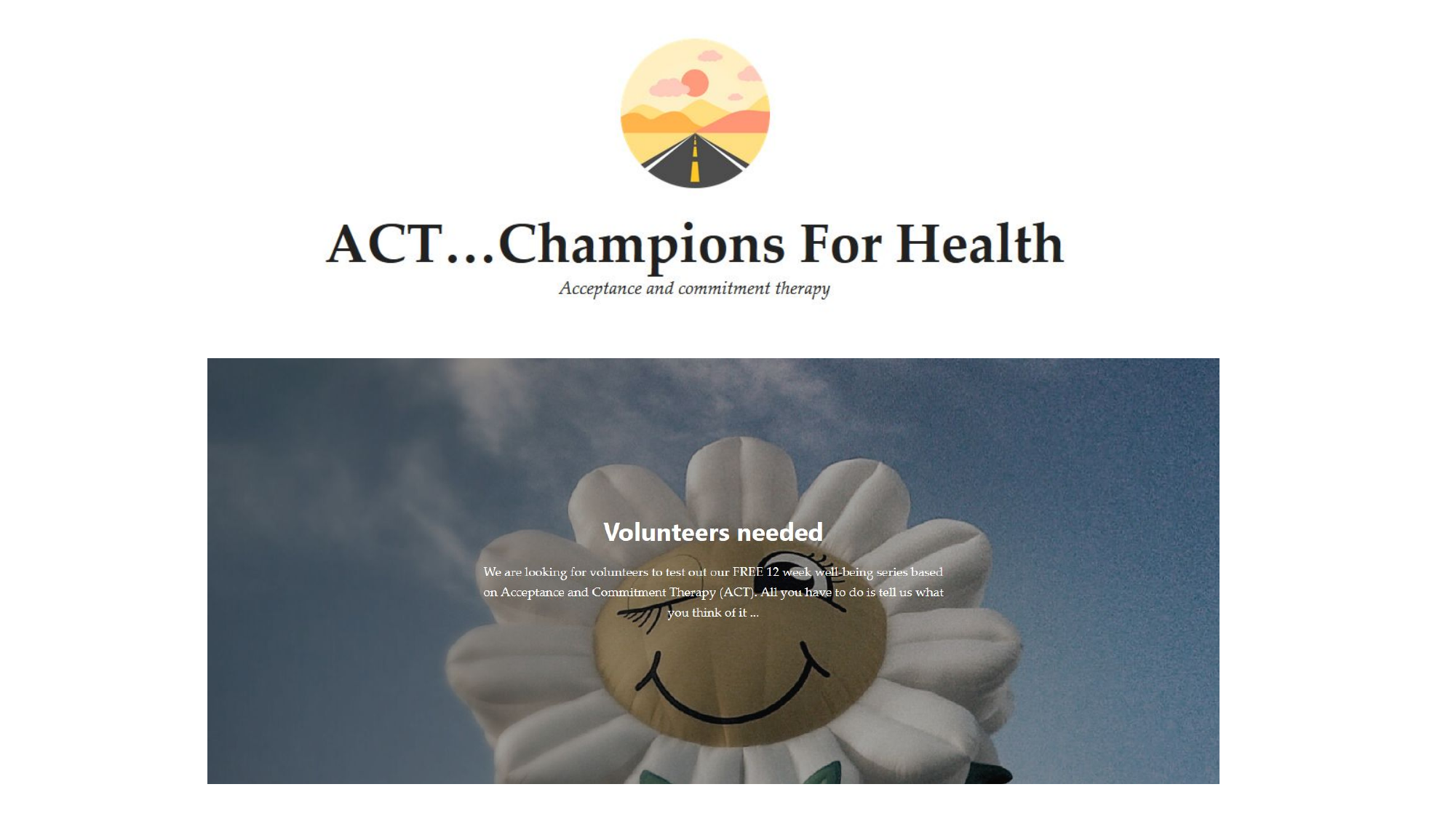

## Slide 2
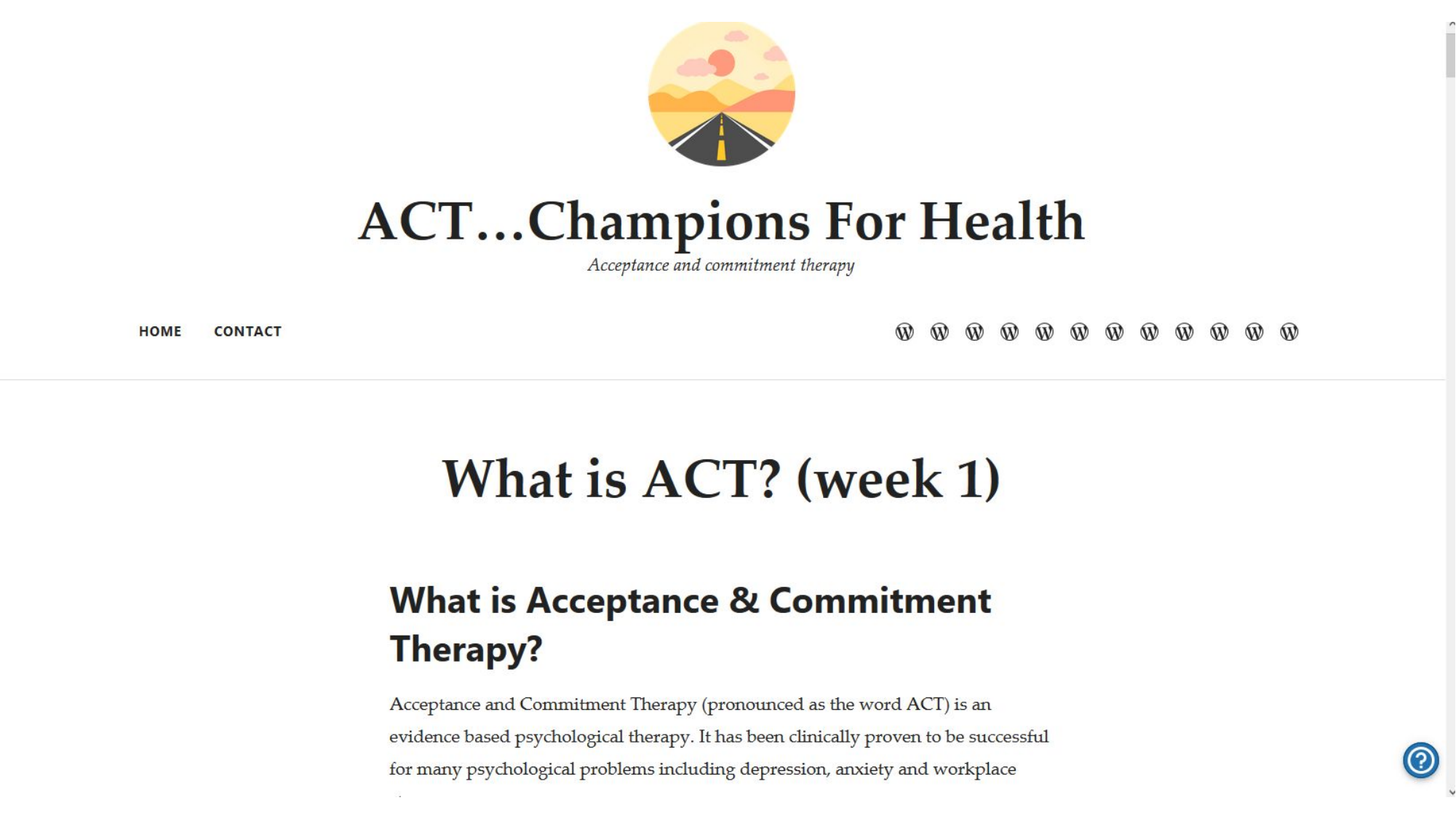

## Slide 3
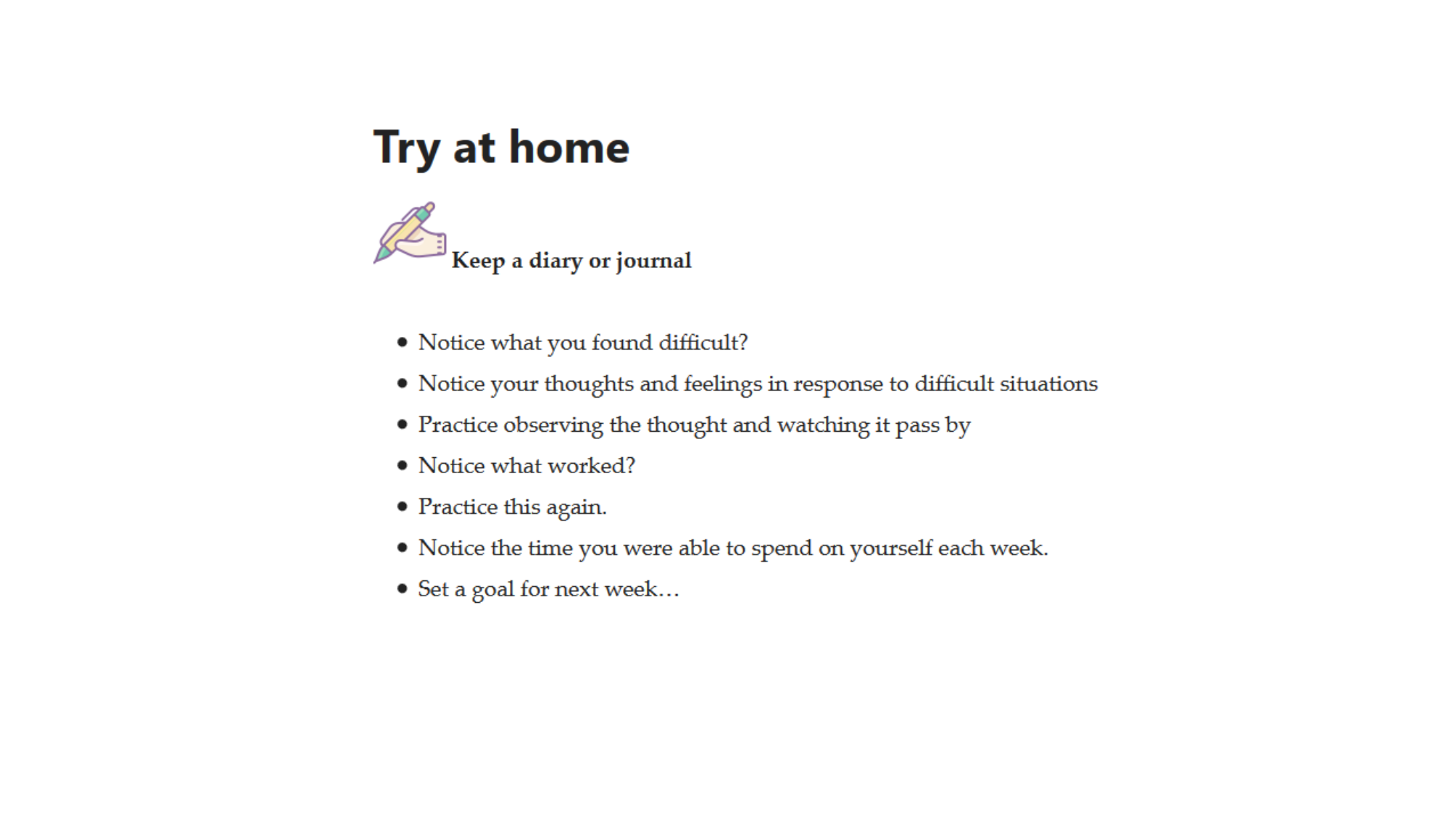

## Slide 4
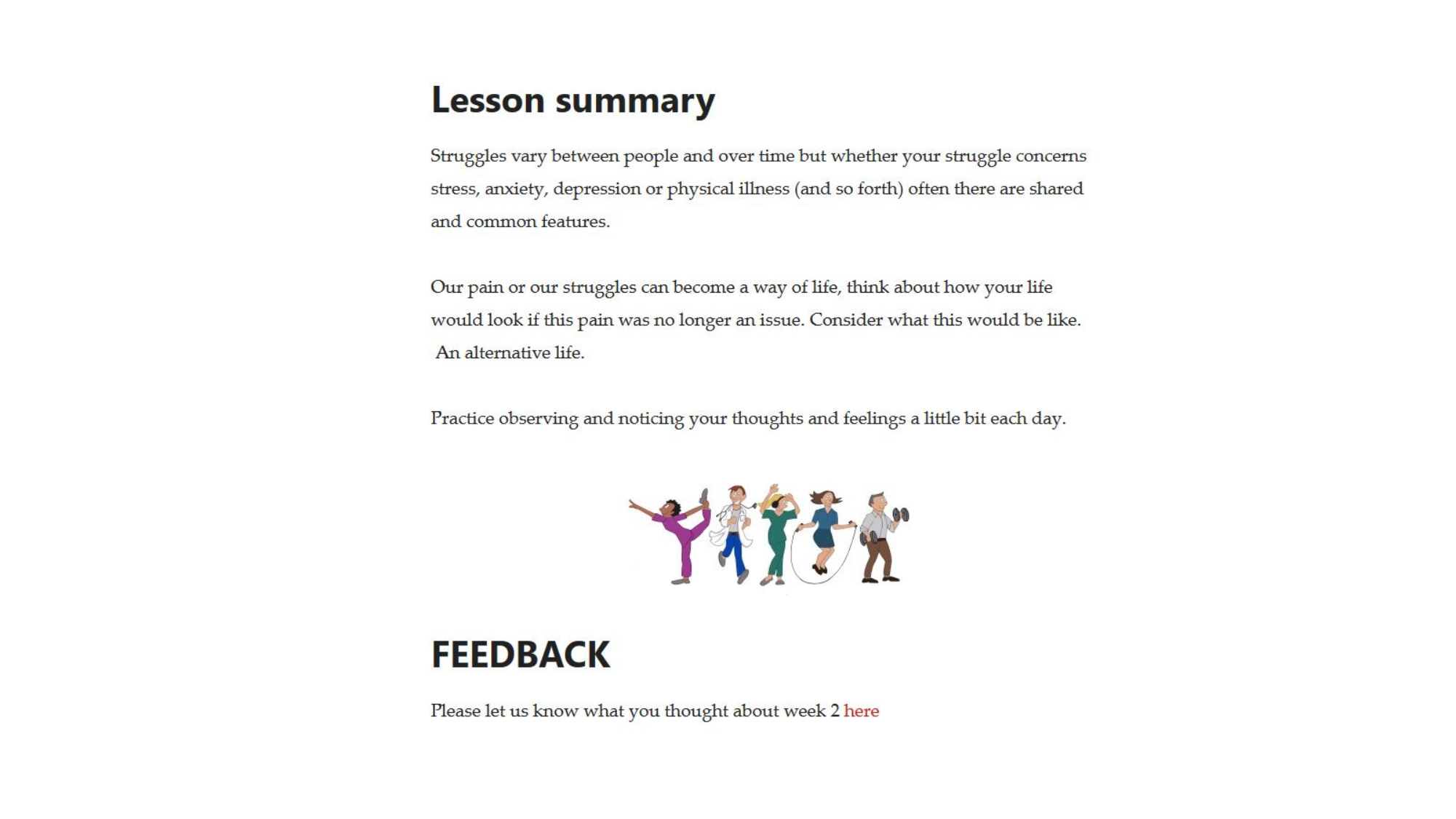

## Slide 5
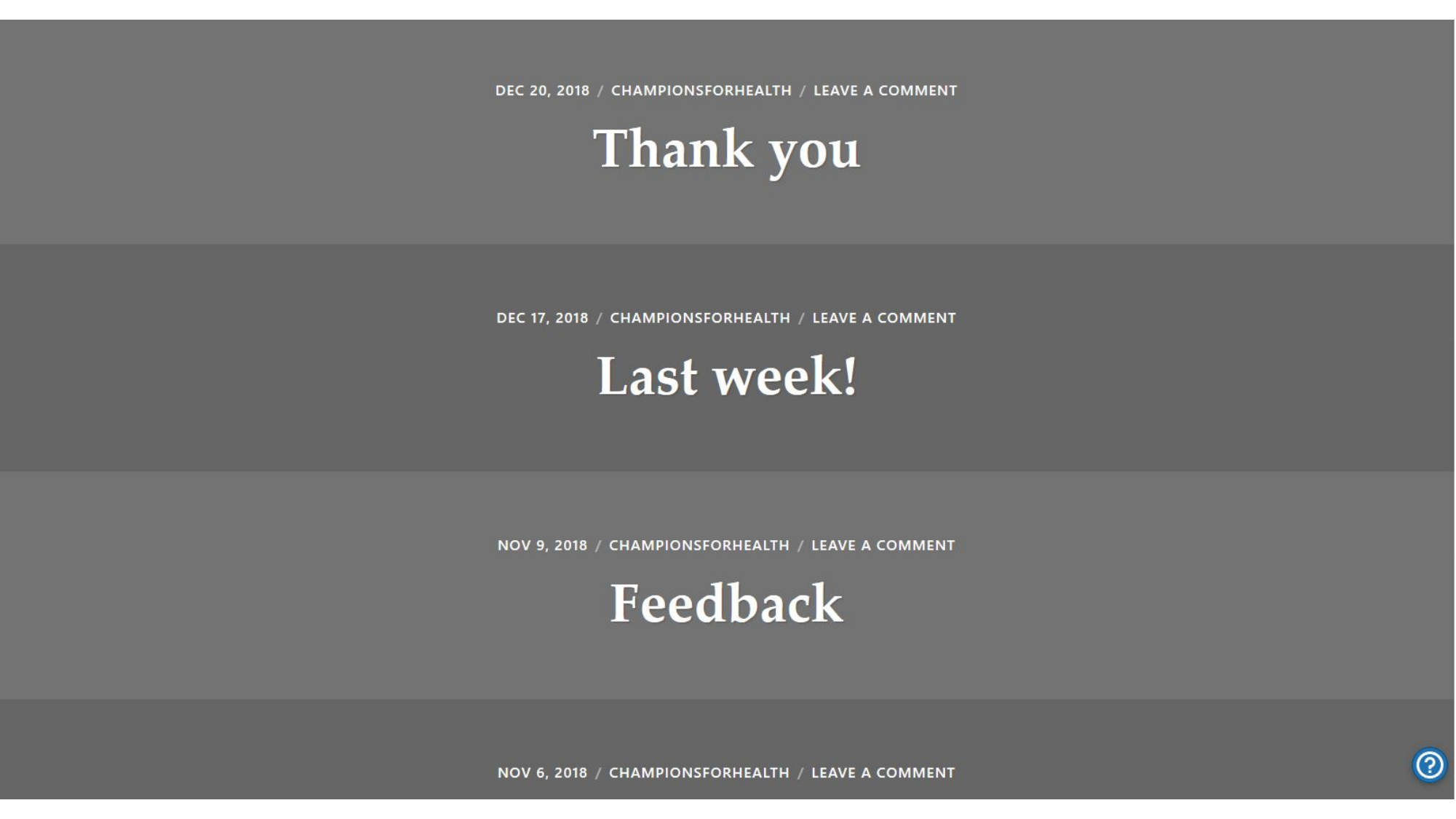

Supplement: Multimedia Appendix 2 [file formative_v4i11e22507_app2.pptx]

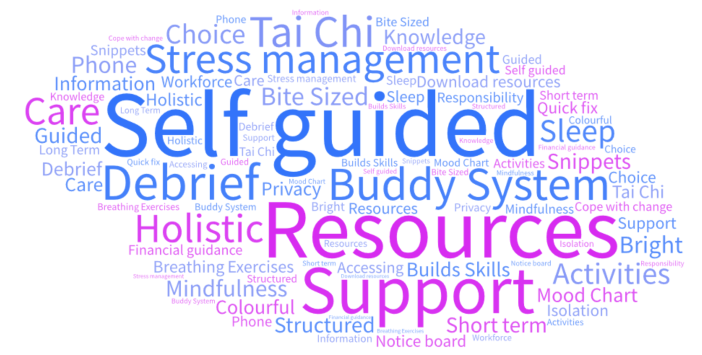

Supplement: Multimedia Appendix 3 [file formative_v4i11e22507_app3.png]
