# Supplementary material for: Development of a Web-Based Acceptance and Commitment Therapy Intervention to Support Lifestyle Behavior Change and Well-Being in Health Care Staff: Participatory Design Study
Source: JMIR Form Res. 2020 Nov 30;4(11):e22507. doi: 10.2196/22507 (PMC7735901; doi:10.2196/22507)
Supplement: Multimedia Appendix 4 [file formative_v4i11e22507_app4.pptx]

## Slide 1
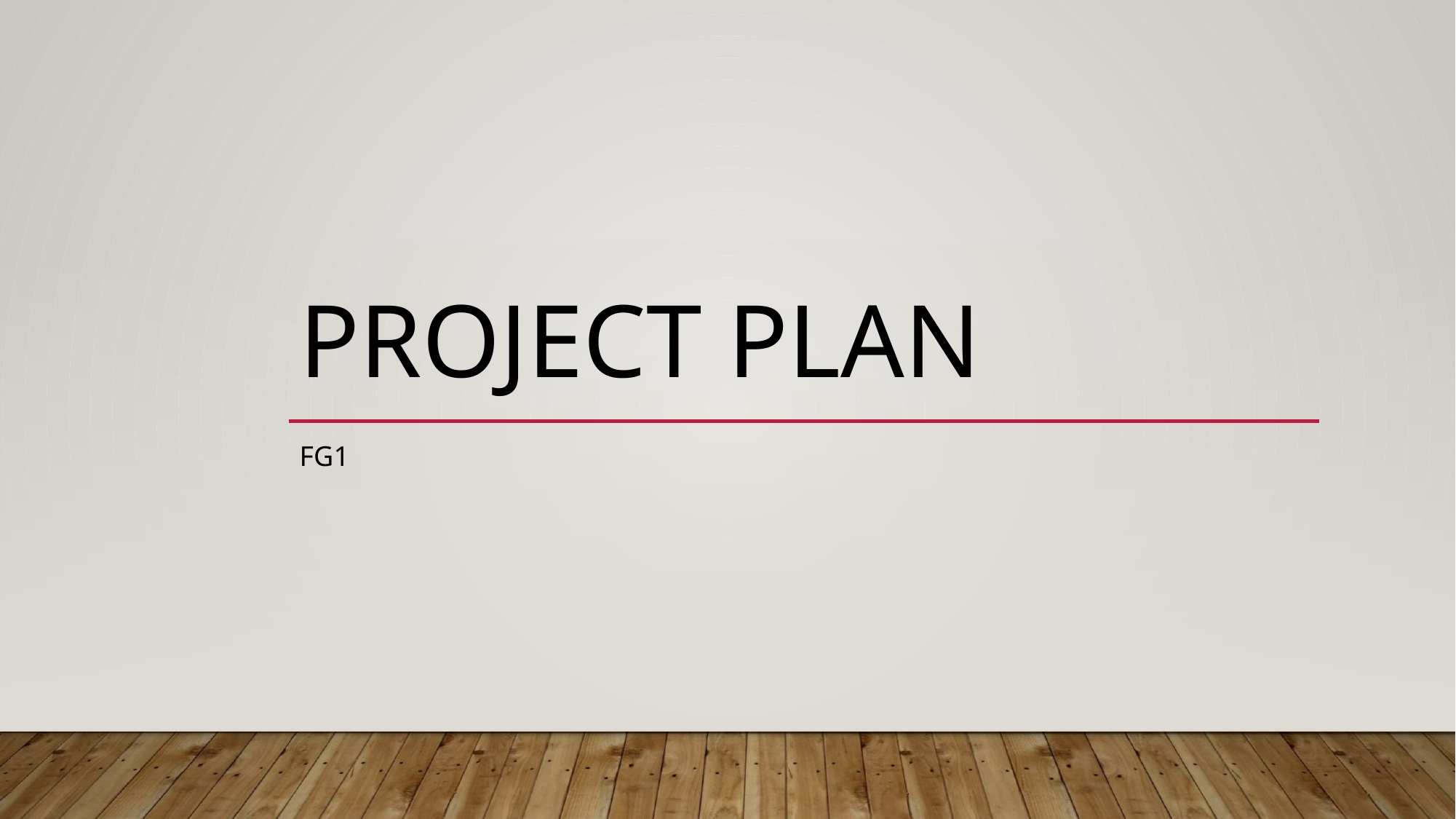

# Project plan
FG1

## Slide 2
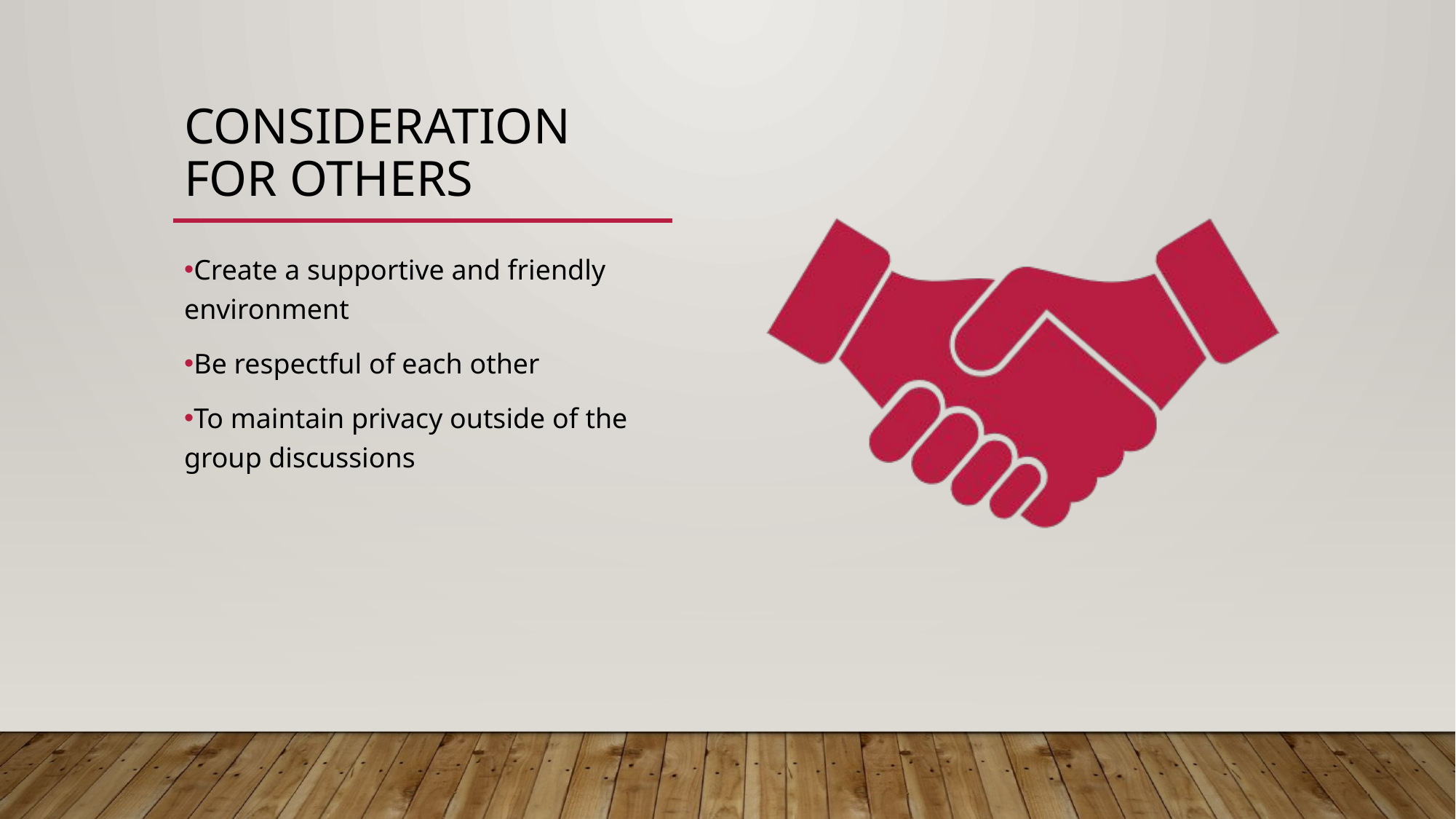

# Consideration for others
Create a supportive and friendly environment
Be respectful of each other
To maintain privacy outside of the group discussions

## Slide 3
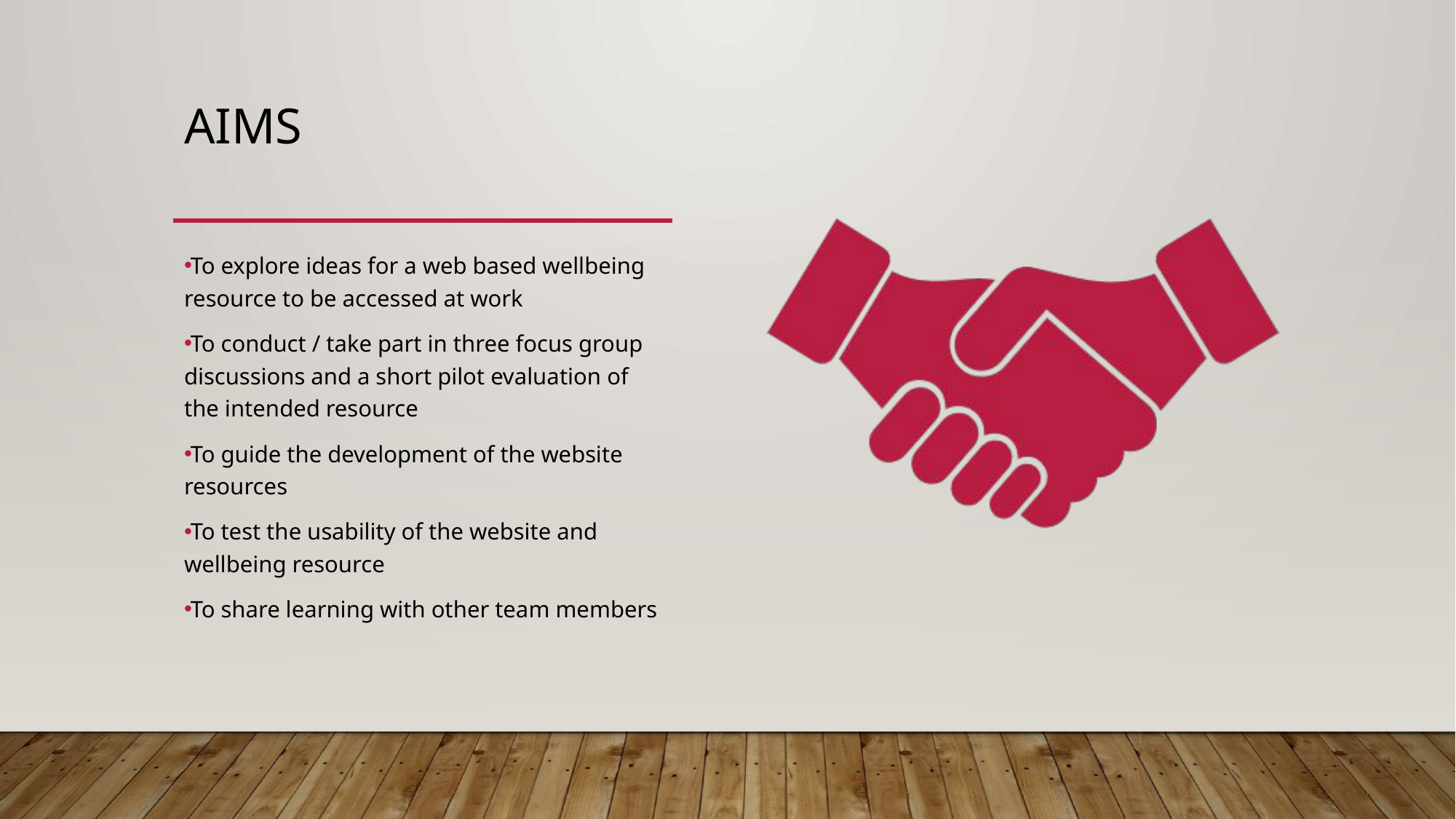

# Aims
To explore ideas for a web based wellbeing resource to be accessed at work
To conduct / take part in three focus group discussions and a short pilot evaluation of the intended resource
To guide the development of the website resources
To test the usability of the website and wellbeing resource
To share learning with other team members

## Slide 4
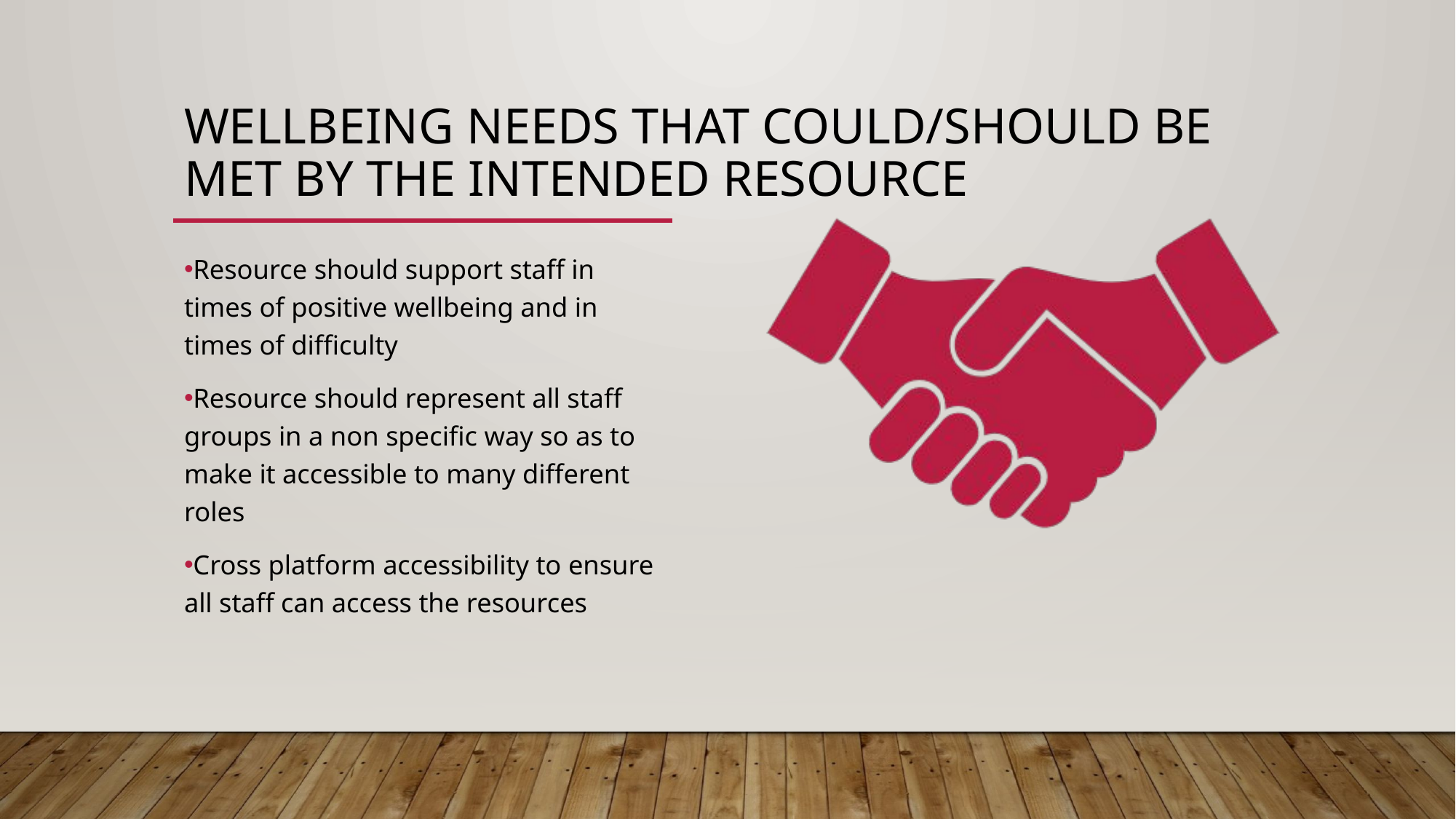

# wellbeing needs that could/should be met by the intended resource
Resource should support staff in times of positive wellbeing and in times of difficulty
Resource should represent all staff groups in a non specific way so as to make it accessible to many different roles
Cross platform accessibility to ensure all staff can access the resources
