# Supplementary material for: Development of a Web-Based Acceptance and Commitment Therapy Intervention to Support Lifestyle Behavior Change and Well-Being in Health Care Staff: Participatory Design Study
Source: JMIR Form Res. 2020 Nov 30;4(11):e22507. doi: 10.2196/22507 (PMC7735901; doi:10.2196/22507)
Supplement: Multimedia Appendix 5 [file formative_v4i11e22507_app5.pptx]

## Slide 1
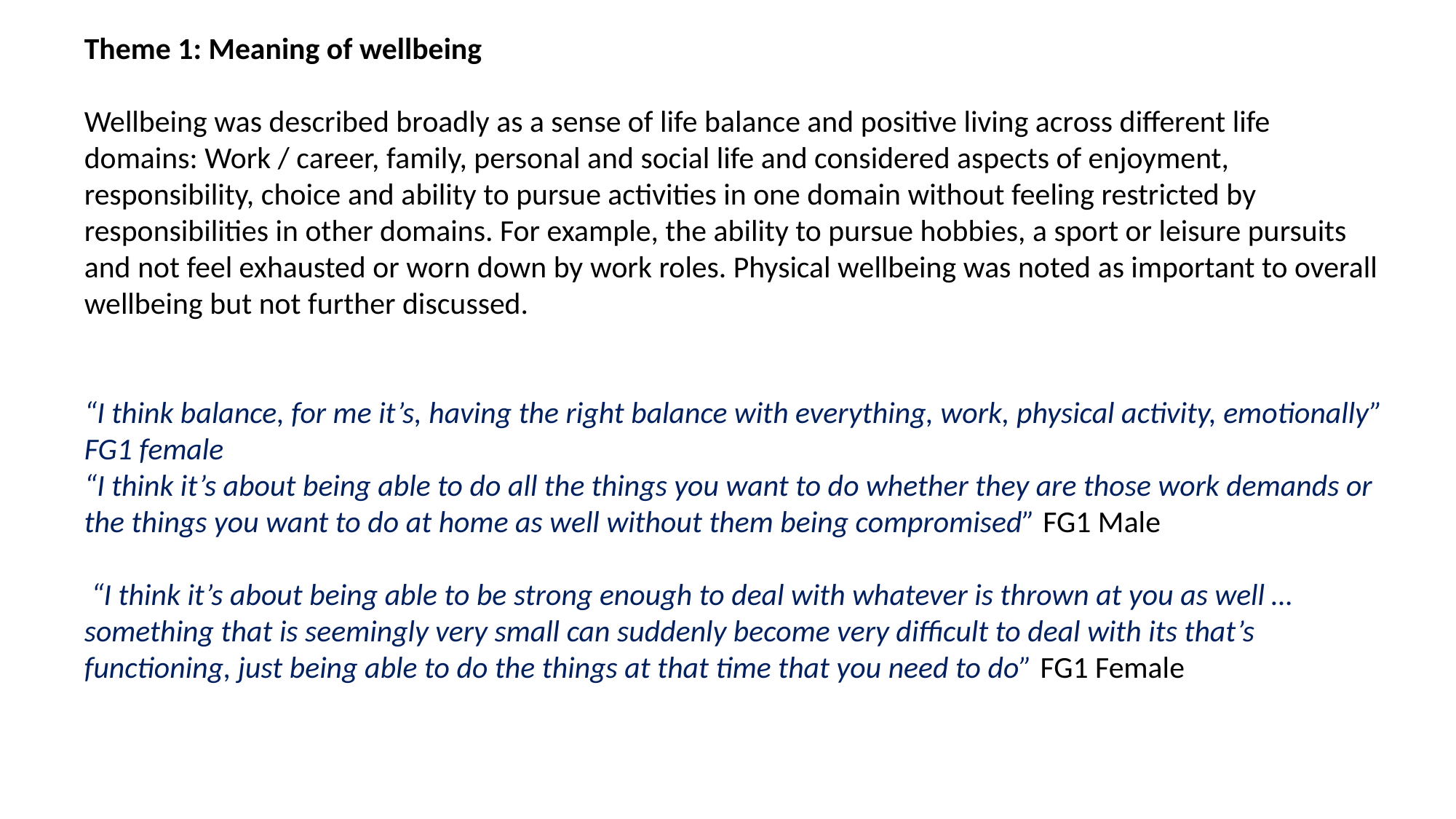

Theme 1: Meaning of wellbeing
Wellbeing was described broadly as a sense of life balance and positive living across different life domains: Work / career, family, personal and social life and considered aspects of enjoyment, responsibility, choice and ability to pursue activities in one domain without feeling restricted by responsibilities in other domains. For example, the ability to pursue hobbies, a sport or leisure pursuits and not feel exhausted or worn down by work roles. Physical wellbeing was noted as important to overall wellbeing but not further discussed.
“I think balance, for me it’s, having the right balance with everything, work, physical activity, emotionally” FG1 female
“I think it’s about being able to do all the things you want to do whether they are those work demands or the things you want to do at home as well without them being compromised” FG1 Male
 “I think it’s about being able to be strong enough to deal with whatever is thrown at you as well …something that is seemingly very small can suddenly become very difficult to deal with its that’s functioning, just being able to do the things at that time that you need to do” FG1 Female

## Slide 2
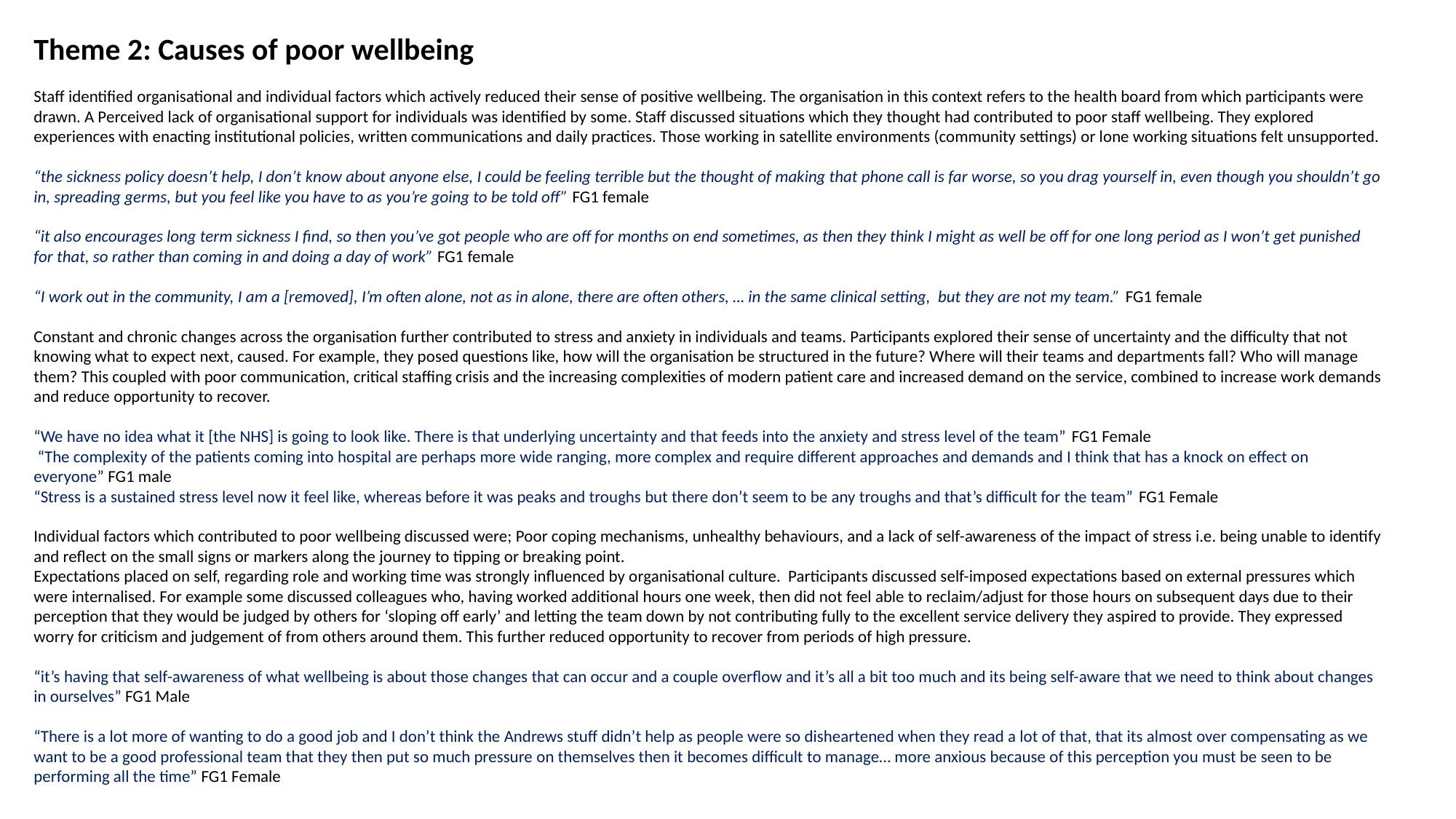

Theme 2: Causes of poor wellbeing
Staff identified organisational and individual factors which actively reduced their sense of positive wellbeing. The organisation in this context refers to the health board from which participants were drawn. A Perceived lack of organisational support for individuals was identified by some. Staff discussed situations which they thought had contributed to poor staff wellbeing. They explored experiences with enacting institutional policies, written communications and daily practices. Those working in satellite environments (community settings) or lone working situations felt unsupported.
“the sickness policy doesn’t help, I don’t know about anyone else, I could be feeling terrible but the thought of making that phone call is far worse, so you drag yourself in, even though you shouldn’t go in, spreading germs, but you feel like you have to as you’re going to be told off” FG1 female
“it also encourages long term sickness I find, so then you’ve got people who are off for months on end sometimes, as then they think I might as well be off for one long period as I won’t get punished for that, so rather than coming in and doing a day of work” FG1 female
“I work out in the community, I am a [removed], I’m often alone, not as in alone, there are often others, … in the same clinical setting, but they are not my team.” FG1 female
Constant and chronic changes across the organisation further contributed to stress and anxiety in individuals and teams. Participants explored their sense of uncertainty and the difficulty that not knowing what to expect next, caused. For example, they posed questions like, how will the organisation be structured in the future? Where will their teams and departments fall? Who will manage them? This coupled with poor communication, critical staffing crisis and the increasing complexities of modern patient care and increased demand on the service, combined to increase work demands and reduce opportunity to recover.
“We have no idea what it [the NHS] is going to look like. There is that underlying uncertainty and that feeds into the anxiety and stress level of the team” FG1 Female
 “The complexity of the patients coming into hospital are perhaps more wide ranging, more complex and require different approaches and demands and I think that has a knock on effect on everyone” FG1 male
“Stress is a sustained stress level now it feel like, whereas before it was peaks and troughs but there don’t seem to be any troughs and that’s difficult for the team” FG1 Female
Individual factors which contributed to poor wellbeing discussed were; Poor coping mechanisms, unhealthy behaviours, and a lack of self-awareness of the impact of stress i.e. being unable to identify and reflect on the small signs or markers along the journey to tipping or breaking point.
Expectations placed on self, regarding role and working time was strongly influenced by organisational culture. Participants discussed self-imposed expectations based on external pressures which were internalised. For example some discussed colleagues who, having worked additional hours one week, then did not feel able to reclaim/adjust for those hours on subsequent days due to their perception that they would be judged by others for ‘sloping off early’ and letting the team down by not contributing fully to the excellent service delivery they aspired to provide. They expressed worry for criticism and judgement of from others around them. This further reduced opportunity to recover from periods of high pressure.
“it’s having that self-awareness of what wellbeing is about those changes that can occur and a couple overflow and it’s all a bit too much and its being self-aware that we need to think about changes in ourselves” FG1 Male
“There is a lot more of wanting to do a good job and I don’t think the Andrews stuff didn’t help as people were so disheartened when they read a lot of that, that its almost over compensating as we want to be a good professional team that they then put so much pressure on themselves then it becomes difficult to manage… more anxious because of this perception you must be seen to be performing all the time” FG1 Female

## Slide 3
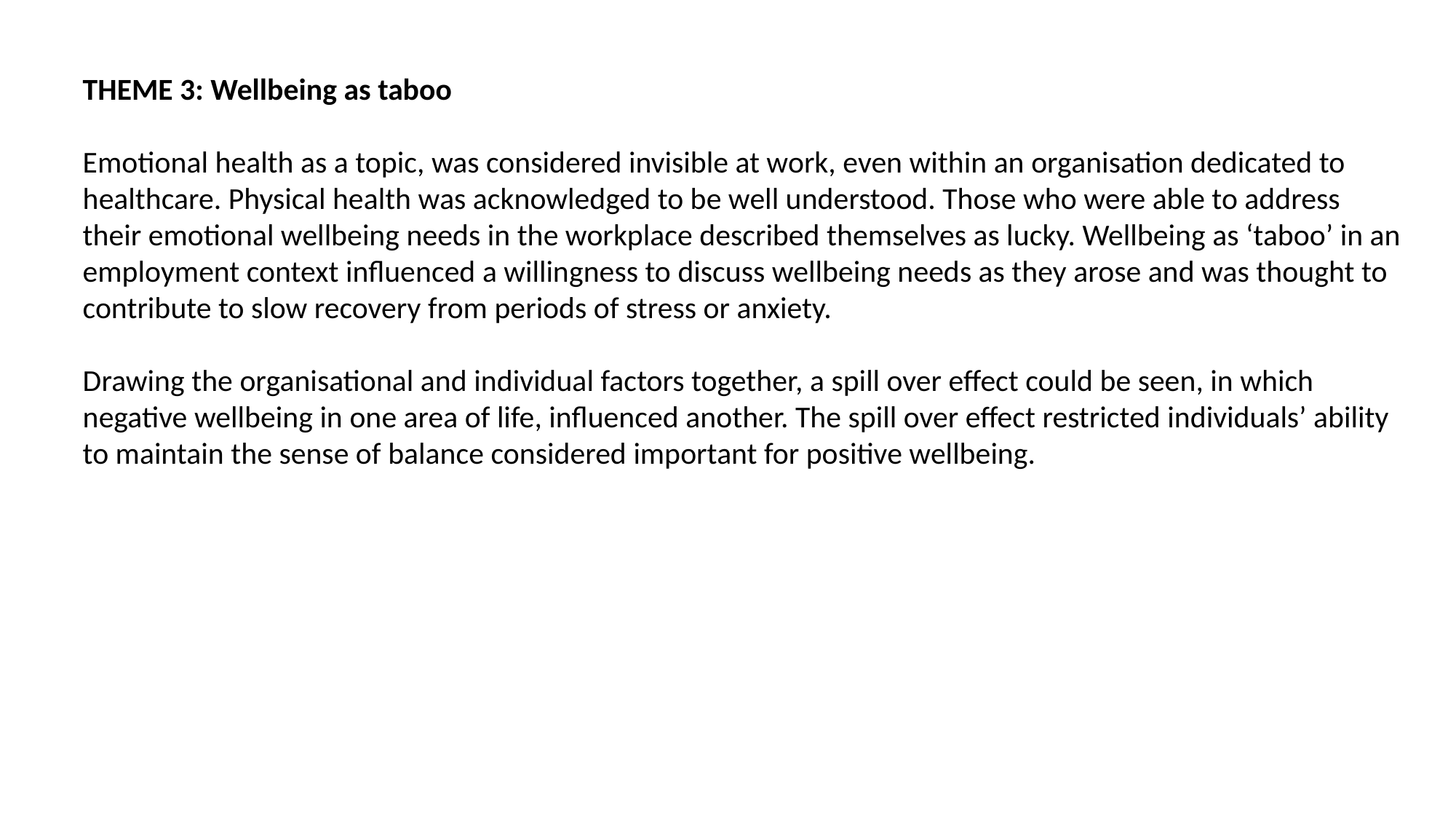

THEME 3: Wellbeing as taboo
Emotional health as a topic, was considered invisible at work, even within an organisation dedicated to healthcare. Physical health was acknowledged to be well understood. Those who were able to address their emotional wellbeing needs in the workplace described themselves as lucky. Wellbeing as ‘taboo’ in an employment context influenced a willingness to discuss wellbeing needs as they arose and was thought to contribute to slow recovery from periods of stress or anxiety.
Drawing the organisational and individual factors together, a spill over effect could be seen, in which negative wellbeing in one area of life, influenced another. The spill over effect restricted individuals’ ability to maintain the sense of balance considered important for positive wellbeing.

## Slide 4
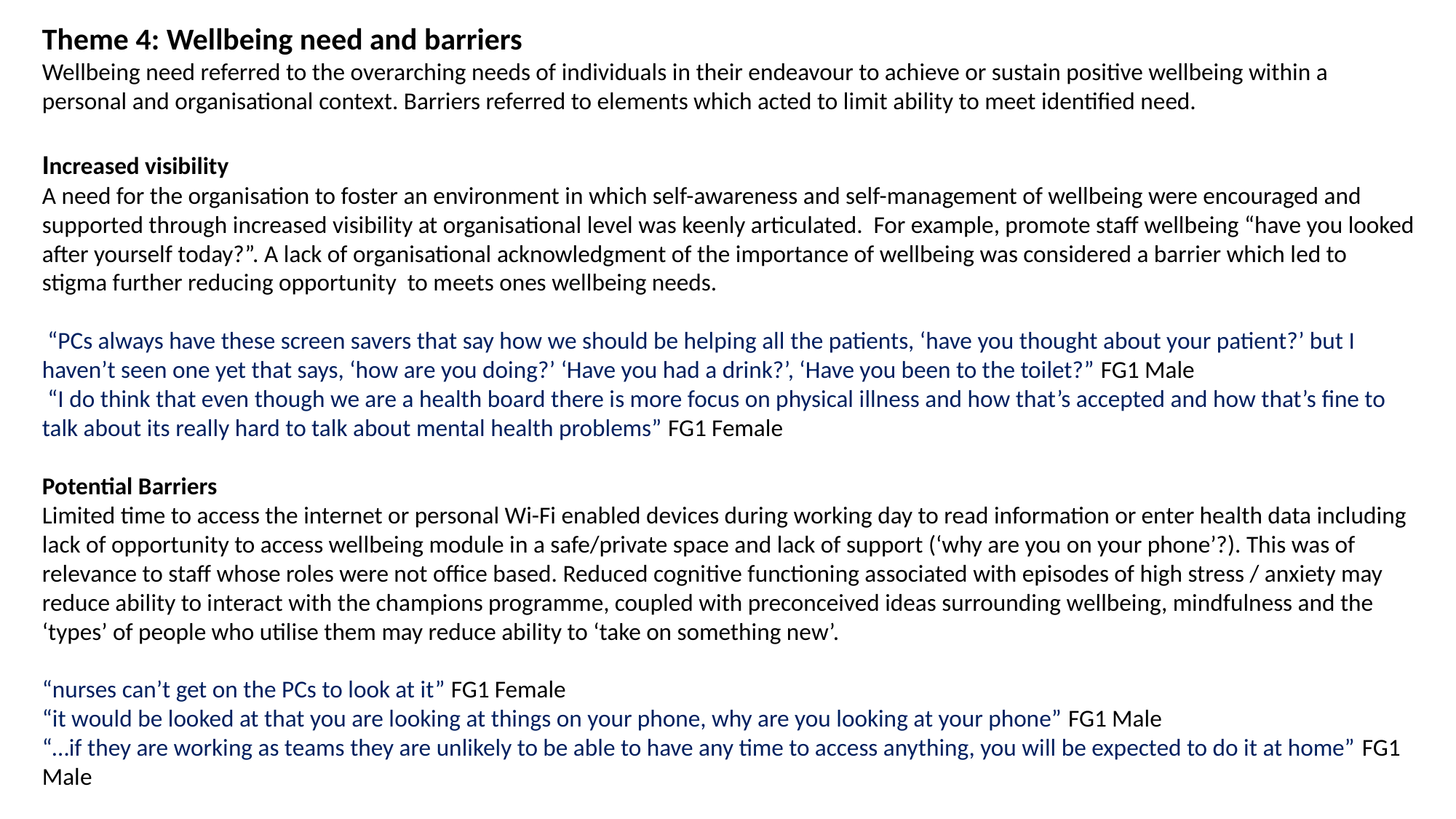

Theme 4: Wellbeing need and barriers
Wellbeing need referred to the overarching needs of individuals in their endeavour to achieve or sustain positive wellbeing within a personal and organisational context. Barriers referred to elements which acted to limit ability to meet identified need.
Increased visibility
A need for the organisation to foster an environment in which self-awareness and self-management of wellbeing were encouraged and supported through increased visibility at organisational level was keenly articulated. For example, promote staff wellbeing “have you looked after yourself today?”. A lack of organisational acknowledgment of the importance of wellbeing was considered a barrier which led to stigma further reducing opportunity to meets ones wellbeing needs.
 “PCs always have these screen savers that say how we should be helping all the patients, ‘have you thought about your patient?’ but I haven’t seen one yet that says, ‘how are you doing?’ ‘Have you had a drink?’, ‘Have you been to the toilet?” FG1 Male
 “I do think that even though we are a health board there is more focus on physical illness and how that’s accepted and how that’s fine to talk about its really hard to talk about mental health problems” FG1 Female
Potential Barriers
Limited time to access the internet or personal Wi-Fi enabled devices during working day to read information or enter health data including lack of opportunity to access wellbeing module in a safe/private space and lack of support (‘why are you on your phone’?). This was of relevance to staff whose roles were not office based. Reduced cognitive functioning associated with episodes of high stress / anxiety may reduce ability to interact with the champions programme, coupled with preconceived ideas surrounding wellbeing, mindfulness and the ‘types’ of people who utilise them may reduce ability to ‘take on something new’.
“nurses can’t get on the PCs to look at it” FG1 Female
“it would be looked at that you are looking at things on your phone, why are you looking at your phone” FG1 Male
“…if they are working as teams they are unlikely to be able to have any time to access anything, you will be expected to do it at home” FG1 Male

## Slide 5
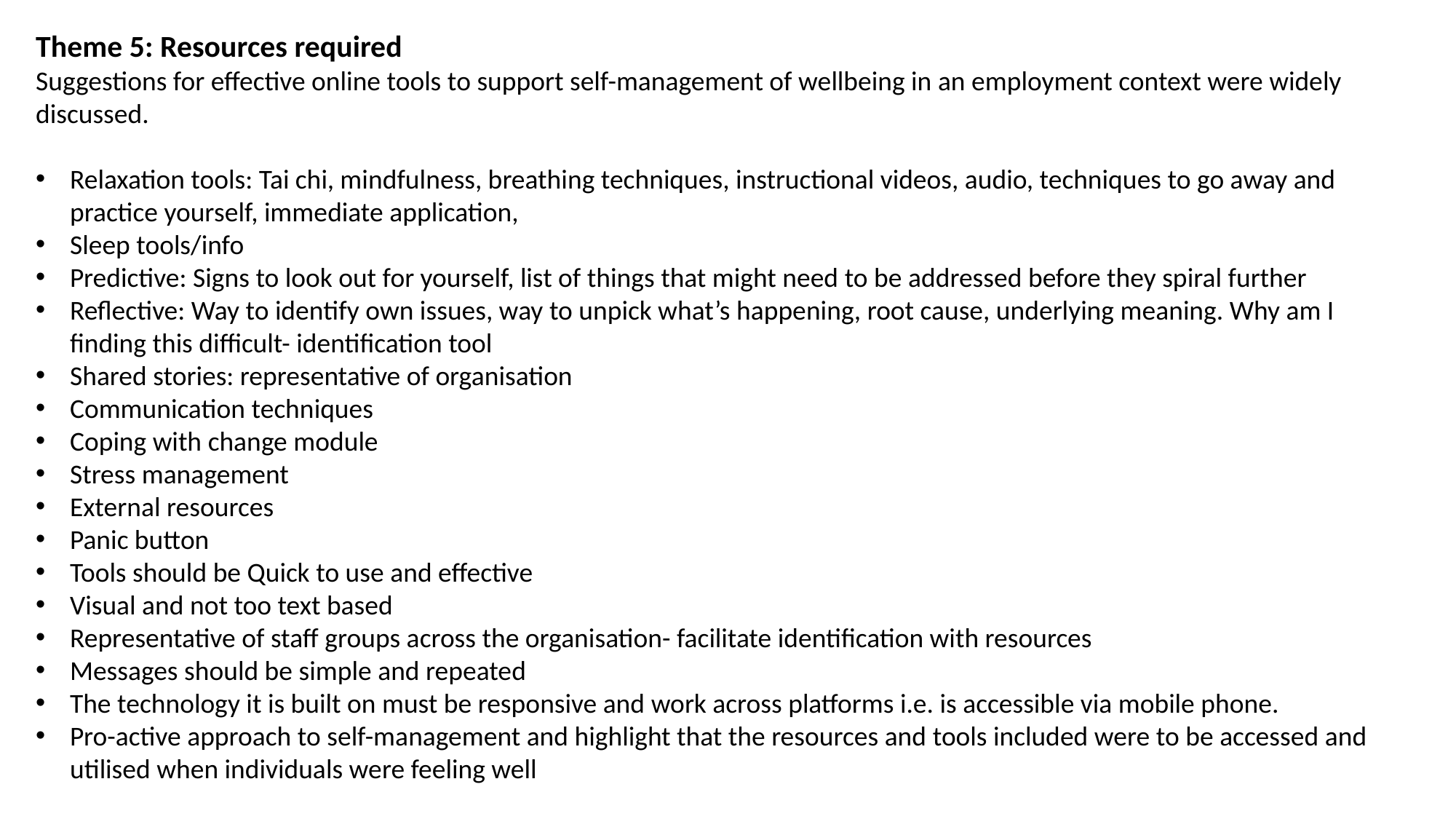

Theme 5: Resources required
Suggestions for effective online tools to support self-management of wellbeing in an employment context were widely discussed.
Relaxation tools: Tai chi, mindfulness, breathing techniques, instructional videos, audio, techniques to go away and practice yourself, immediate application,
Sleep tools/info
Predictive: Signs to look out for yourself, list of things that might need to be addressed before they spiral further
Reflective: Way to identify own issues, way to unpick what’s happening, root cause, underlying meaning. Why am I finding this difficult- identification tool
Shared stories: representative of organisation
Communication techniques
Coping with change module
Stress management
External resources
Panic button
Tools should be Quick to use and effective
Visual and not too text based
Representative of staff groups across the organisation- facilitate identification with resources
Messages should be simple and repeated
The technology it is built on must be responsive and work across platforms i.e. is accessible via mobile phone.
Pro-active approach to self-management and highlight that the resources and tools included were to be accessed and utilised when individuals were feeling well

## Slide 6
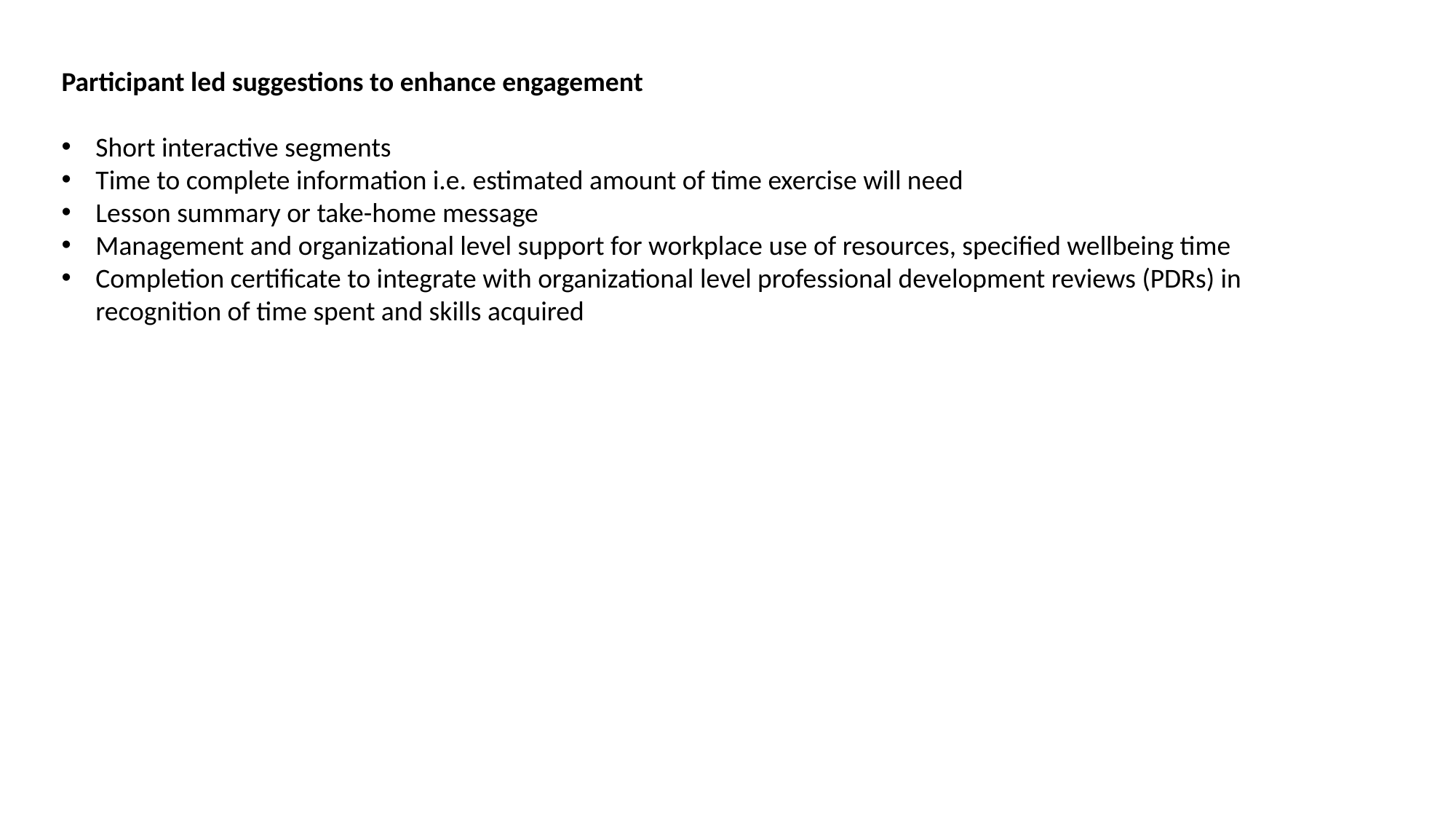

Participant led suggestions to enhance engagement
Short interactive segments
Time to complete information i.e. estimated amount of time exercise will need
Lesson summary or take-home message
Management and organizational level support for workplace use of resources, specified wellbeing time
Completion certificate to integrate with organizational level professional development reviews (PDRs) in recognition of time spent and skills acquired
